# Are Machine Learning Models on Wrist Accelerometry Robust against Differences in Physical Performance among Older Adults?

**DOI:** 10.3390/s22083061

**Published:** 2022-04-15

**Authors:** Chen Bai, Amal A. Wanigatunga, Santiago Saldana, Ramon Casanova, Todd M. Manini, Mamoun T. Mardini

**Affiliations:** 1 Department of Health Outcomes and Biomedical Informatics, College of Medicine, University of Florida, Gainesville, FL 32610, USA; tmanini@ufl.edu (T.M.M.); malmardini@ufl.edu (M.T.M.); 2 Department of Epidemiology, Bloomberg School of Public Health, Johns Hopkins University, Baltimore, MD 21205, USA; awaniga1@jhu.edu; 3 Department of Biostatistics and Data Science, School of Medicine, Wake Forest University, Winston-Salem, NC 27101, USA; ssaldana@wakehealth.edu (S.S.); casanova@wakehealth.edu (R.C.)

**Keywords:** wrist, accelerometer, short physical performance battery, physical activity, energy expenditure, eXtreme Gradient Boosting

## Abstract

Sufficient physical activity (PA) reduces the risk of a myriad of diseases and preserves physical capabilities in later life. While there have been significant achievements in mapping accelerations to real-life movements using machine learning (ML), errors continue to be common, particularly for wrist-worn devices. It remains unknown whether ML models are robust for estimating age-related loss of physical function. In this study, we evaluated the performance of ML models (XGBoost and LASSO) to estimate the hallmark measures of PA in low physical performance (LPP) and high physical performance (HPP) groups. Our models were built to recognize PA types and intensities, identify each individual activity, and estimate energy expenditure (EE) using wrist-worn accelerometer data (33 activities per participant) from a large sample of participants (*n* = 247, 57% females, aged 60+ years). Results indicated that the ML models were accurate in recognizing PA by type and intensity while also estimating EE accurately. However, the models built to recognize individual activities were less robust. Across all tasks, XGBoost outperformed LASSO. XGBoost obtained F1-Scores for sedentary (0.932 ± 0.005), locomotion (0.946 ± 0.003), lifestyle (0.927 ± 0.006), and strength flexibility exercise (0.915 ± 0.017) activity type recognition tasks. The F1-Scores for recognizing low, light, and moderate activity intensity were (0.932 ± 0.005), (0.840 ± 0.004), and (0.869 ± 0.005), respectively. The root mean square error for EE estimation was 0.836 ± 0.059 METs. There was no evidence showing that splitting the participants into the LPP and HPP groups improved the models’ performance on estimating the hallmark measures of physical activities. In conclusion, using features derived from wrist-worn accelerometer data, machine learning models can accurately recognize PA types and intensities and estimate EE for older adults with high and low physical function.

## 1. Introduction

Accurate prediction of physical activity (PA) type, intensity, and duration is critical for estimating an individual or population’s accumulation of PA and measuring achievements in reaching both national and international recommended levels. The rapid growth of fitness trackers and smartwatches with built-in accelerometers provides an objective method to achieve this goal. While there have been significant achievements in finding meaningful patterns in accelerometry data using machine learning [1,2,3,4,5,6,7,8,9,10,11], errors continue to be common, particularly for wrist-worn devices. The wrist position is not biomechanically suited for the estimation of many types of PA. Previous studies have utilized machine learning (ML) models to process and model accelerometer data, including support vector machine [1,2], random forest [2,4], deep neural network [4,5], and other models [6,7,8,9,10,11]. However, whether these machine learning models are robust for estimating age-related loss of physical function remains unknown.

The participant sample used to build the ML models will greatly influence their capability to generalize to the broader population. In that regard, there has been little understanding of whether ML models are robust to demographic and physical health differences. While our previous work has expanded knowledge about age differences (three age groups have been explored, which are young [20–50], middle (50–70], and old (70, 89] years) [12], there remains a paucity of work on older adults [13,14]. It remains unknown whether deteriorated physical performance due to the aging process, chronic health conditions, and other possible factors influences the ML models’ performance in estimating PA type and intensity from wrist-worn accelerometers. Older adults with low physical function move slower [15,16], with more variability [17,18], and seek assistance using their upper body to perform daily tasks. These modifications would all influence their tri-axial accelerometer output. As a result, ML methods may need to account for variations in physical function status to enhance their accuracy in estimation.

**Figure 1:**
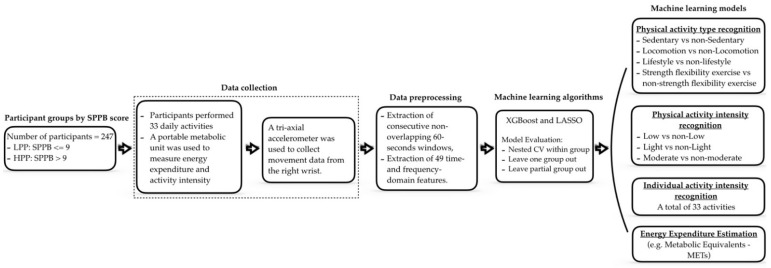
A flow diagram of the steps followed to collect and process the accelerometer data.

We hypothesized that older adults with low physical function reduce the performance of ML models on estimating hallmark metrics of PA, including types, intensities, and energy expenditure (EE). Our main contribution was to examine the effect of age-related loss of physical function on the performance of ML models across older adults with low and high physical performance. To the best of our knowledge, this is the first attempt to examine the robustness of ML models in this aspect. Our demonstrated outcomes were based on several experiments that included: (1) examining distinct ML models trained and tested on participants from different performance groups (low performance and high performance groups); (2) examining ML models trained on one group and tested on the other (e.g., training on the high performance group and testing on the low performance group, and vice versa); and (3) examining ML models trained on both performance groups and tested on a subgroup of one of the performance groups (e.g., training on low performance group and 50% of high performance group, and testing on the remaining 50% high performance group). We utilized a large dataset of wrist-worn accelerometer data coupled with energy expenditure (EE) measurements from 247 high- and low-functioning older adults who performed 33 standardized activities in a laboratory setting. A validated physical performance test was used to rank individuals and place them into either low or high function groups [19]. Figure 1 illustrates the overall steps followed to collect and process the data and the machine learning models built.

## 2. Materials and Methods

In this section, we first present the characteristics of the participants and explain the criterion adopted to split participants into high and low physical performance groups. After that, we describe the physical activities performed by participants and the instruments used to capture the accelerometer and energy expenditure data. Finally, we formulate the research problem and explain the machine learning models that we built to test our hypothesis.

### 2.1. Participants and Short Physical Performance Battery

We previously described the ChoresXL and PEAKS study methods in detail [20,21]. Briefly, data from the ChoresXL [20] (*n* = 246) and PEAKS [21] (*n* = 129) studies were used for this analysis. Participants from both studies had identical eligibility criteria. They were community-dwelling adults aged 60+ years who could read and speak English and had a stable weight (+/−5 lbs) for the prior three months. Participants were excluded from the study if they had a severe disability or used a walker, had evidence of significant cognitive impairment, a known neuromuscular disorder (e.g.,Parkinson’s disease, Myasthenia Gravis, or post-stroke ambulatory deficits), significant pain that would impair movement, sensory impairment that would preclude study assessment, severe lung disease requiring supplemental oxygen, severe cardiac disease (NYHA Class II or IV congestive heart failure, aortic stenosis), or other chronic health conditions that would impact safety or the study protocol (e.g., on dialysis, schizophrenia, or use of anabolic medications). Participants or their specific activity data were excluded from the analysis in this study if there were missing start/end times for activities (25 participants), lacked demographic information or physical function (6 participants), or had insufficient activity length or experienced technical issues (3 participants). All study procedures were approved by the University of Florida Institutional Review Board. Written informed consent was obtained from all participants before the study. 

The Short Physical Performance Battery (SPPB) was used to quantify lower extremity function [19] and categorize functional groups. The battery includes a timed 4 m walk, 5 repeated chair stands, and 3 balance tests. Each is evaluated on a scale of 0 (worst) to 4 (best) derived from population-based normative data. Each test is used to create a summary SPPB score that ranges from 0 (worst performers) to 12 (best performers) [22]. The SPPB has excellent test-retest reliability and demonstrates construct validity by predicting institutionalization, mortality, and disability [23]. Consistent with our previous work [24,25], we categorized participants with an SPPB ≤ 9 as having low physical performance (LPP) and those with an SPPB > 9 as having high physical performance (HPP). 

### 2.2. Standardized Activities

Participants completed a battery of 33 typical daily activities, which were divided into activity types and their intensities were calculated post-facto using metabolic unit data. The tasks were chosen because they mimicked daily chores and activities, common among most Americans, and corresponded to the average time spent in the 2010 American Time Use Survey [26]. All tasks were completed in a standardized laboratory environment by following scripted instructions continuously for approximately 8–10 min to obtain steady-state energy expenditure. Participants completed all tasks at their own pace and the tasks were performed from lowest to highest metabolic demand to minimize task-to-task transfer of residual metabolic effects. Participants completed the 33 tasks over four visits to alleviate exhaustion and ease the burden. Overall, there were 838 data collection visits made in total. Some participants completed all four visits (*n* = 152, 61.5%) and others completed three (*n* = 57, 23.1%), two (*n* = 21, 8.5%) or one (*n* = 17, 6.9%) visits. All data collected that passed quality metrics, regardless of whether participants completed all visits, were used in our analyses. 

### 2.3. Instrumentation

Participants were instructed to wear an ActiGraph GT3X-BT monitor on their right wrist (ActiGraph Inc, Pensacola, FL, USA). The ActiGraph GT3X-BT monitor is a tri-axial lightweight accelerometer that measures acceleration in units of gravity (1 g) in 3D axes. Accelerometers were configured to collect data at a sampling rate of 100 Hz. Additionally, participants wore a portable metabolic unit weighing 2 Kg which calculated energy expenditure using principles of indirect calorimetry (Cosmed K5, COSMED, Rome, Italy). Prior to data collection, the oxygen (O_2_) and carbon dioxide (CO_2_) sensors were calibrated using a gas mixture sample of 16.0% O_2_ and 5.0% CO_2_ and room air calibration. With the use of a 3.0 L syringe, the turbine flow meter was calibrated. A flexible facemask was connected to the flow meter and placed over the participant’s mouth and nose. Oxygen consumption (VO_2_ = mL·min^−1^·kg^−1^) was measured breath-by-breath and was then smoothed using a 30 s running average window. For each activity, steady-state VO_2_ was manually estimated over a ~2 min window where plateau was observed; this state indicates that metabolic demand is matched to physical workload and is an appropriate estimate of energy expenditure for a given task. VO_2_ (mL·min^−1^·kg^−1^) measurements were divided by 3.5 to express data as metabolic equivalents (METs) [27].

### 2.4. Problem Formulation

Hallmark measures of PA were summarized under three classification tasks (recognizing PA type, recognizing PA intensity, and recognizing individual PAs) and one regression task (estimating the energy expenditure). PA types were categorized into four binary classifications: (i) sedentary vs. non-sedentary; (ii) locomotion vs. non-locomotion, (iii) lifestyle vs. non-lifestyle; and (iv) strength flexibility exercise (SFE) vs. non-strength flexibility exercise. PA intensities were categorized into three binary classifications: (i) low vs. non-low; (ii) light vs. non-light; and (iii) moderate vs. non-moderate. 

Wrist accelerometer data were split into non-consecutive 60 s windows that were used to extract time- and frequency-domain features. The window size selection was based on a balance between having sufficient data for effective and stable feature extraction. In total, we extracted 49 time and frequency–domain features chosen from previous literature and our own research [12,28,29,30,31,32] as listed in Table 1. 

### 2.5. Model Training

To build our machine learning models, we applied two machine learning algorithms: eXtreme Gradient Boosting (XGBoost) and Least Absolute Shrinkage and Selection Operator (LASSO). XGBoost is an ensemble learning algorithm in which models are developed sequentially in order to increase (boost) the performance of the prior models by using gradient descent to minimize the errors [33]. LASSO is a statistical algorithm commonly used for feature selection [34,35]. We chose both models due to their better interpretability and inclusion of feature selection throughout the model-building process. The analysis incorporated data from all participants and activities (33 activities). In the estimation of EE, we included 220 participants who provided valid data. Fifty-one machine learning models were built to recognize the tasks mentioned above for the LPP group, the HPP group, and the cohort of all participants (combining the LPP and HPP). Twenty-four models were built to recognize PA type and 18 models were built to recognize PA intensity. To recognize individual PAs, we built three multi-class classification models for each physical performance group using only XGBoost. Finally, six regression models were built to estimate EE. 

Both machine learning algorithms used in this study are naturally resistant to features that have only trivial effects on the prediction. They select important features intrinsically to improve the performance of the models [36]. To address data imbalance issues, the models were designed to automatically adapt weights inversely proportional to the frequency of the classes in the input data. To evaluate the machine learning algorithms, we performed nested cross-validation with five outer folds and five inner folds. One-fifth of the participants served as a test set in each outer fold of the nested cross-validation, and the remaining four-fifths of the participants served as a training set. The outer training set was then divided into five inner folds, each of which functioned as an independent validation set, while the remaining four inner folds served as the training set. Each participant would only be assigned to either the training or test (or validation) set in each inner and outer fold. The inner loop was in charge of hyperparameter tuning (finding the optimal parameters of the model). Additionally, the error estimation and generalization were performed in the outer loop. This procedure was carried out five times. Each time, the model with the highest performance was chosen, and the final performance of the algorithm was reported by averaging the performance of the five outer testing datasets. This approach incorporated model selection into the model fitting process, hence avoiding the bias in performance evaluation [37,38,39]. Although cross-validation is unnecessary in ensemble learning such as XGBoost, we opted to apply it for consistency with the other method (LASSO) [40]. We reported the models’ performance using four metrics: F1-Score, area under the curve (AUC), balanced accuracy, and accuracy. The root mean square error (RMSE) was used to evaluate performance for continuous data from energy expenditure (METs). 

Leaving one (group) out (LOO) and leaving partial (group) out (LPO) using XGBoost for recognition of activity type and intensity, EE estimation tasks were conducted to investigate the effect of the physical function on the performance of ML models across the LPP and HPP groups. LOO was achieved by training purely on participants from one group (e.g., LPP) and evaluating on participants from the other group (e.g., HPP). LPO was achieved by training on a combination of participants from one group and a random sample of 50% of the participants from the other group (e.g., LPP + 50% HPP), and evaluating on the remaining 50% participants (e.g., 50% HPP). LOO and LPO were evaluated using five-fold cross-validation and F1-Scores were reported by evaluating the best models on the test set. In addition, the LPO for each task was repeated 10 times and the average F1-Scores were reported to minimize the selection bias.

## 3. Results

Participants’ descriptive characteristics for each physical performance group are presented in Table 2. Participants from the LPP group have higher average age (75.9 ± 6.6 vs. 70.3 ± 6.6 years) and BMI (30.8 ± 8.8 vs. 27.5 ± 4.8 kg/m^2^), higher rate of chronic health conditions (reported as the percentage of participants who have the condition in each group), and higher average disease index (defined as the number of chronic health conditions), but slower average walk speed (0.79 ± 0.15 vs. 1.05 ± 0.17 m/s) than participants from the HPP group. 

**Figure 2:**
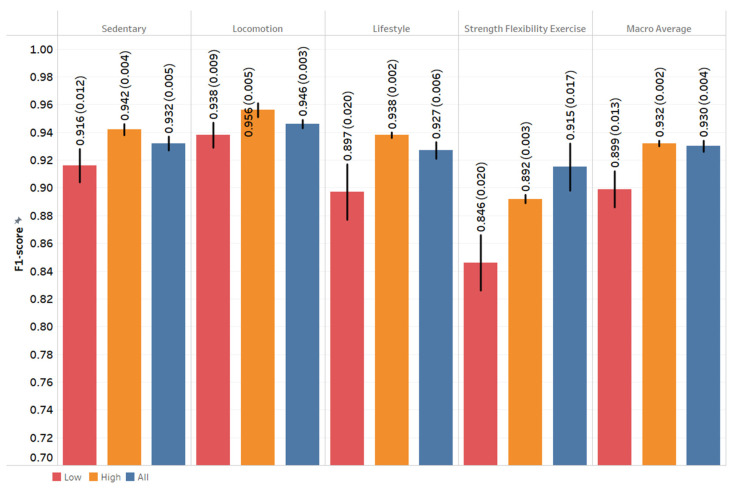
The F1-Score of physical activity type recognition task using XGBoost. Each value is the mean and standard deviation of the five-fold nested cross-validation. Low, high, and all groups represent models built for the low physical performance group, high physical performance group, and all cohort, respectively.

Figure 2 shows that the XGBoost models built on data from the HPP group had slightly better performance than the models built on data from the LPP group in PA type recognition. For both groups, models built to recognize locomotion activities had the highest performance. Models built to recognize sedentary and lifestyle activities achieved similar performance, while models built to recognize strength flexibility exercise (SFE) activities resulted in the lowest performance for both groups. Across all models, the XGBoost algorithm outperformed the LASSO logistic regression. Figures presented in the results section are from XGBoost. The performance metrics of LASSO regression can be found in the Supplementary Materials. Other performance metrics including AUC, accuracy, and balanced accuracy are shown in Figures S1–S7 (see Supplementary Materials).

Figure S8 shows the comparison of F1-Scores of PA type recognition tasks using leave one (group) out (LOO) and leave partial (group) out (LPO) for the LPP and HPP groups, respectively. Training on one group and testing on the other group resulted in a slight difference in performance. There was a slight decrease when training on the LPP group and testing on the HPP group. 

**Figure 3:**
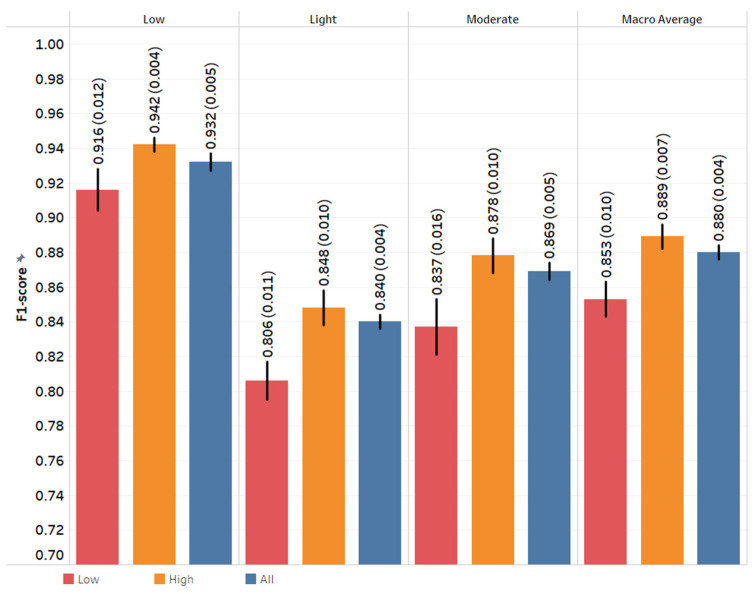
The F1-Score of physical activity intensity recognition task using XGBoost. Each value is the mean and standard deviation of the five-fold nested cross-validation. Low, high, and all groups represent models built for low physical performance group, high physical performance group, and all cohort, respectively.

In PA intensity recognition, the performance of the XGBoost models was slightly higher for the HPP group than the LPP group, as shown in Figure 3. The models of low intensity outperformed the models of moderate, then light intensity. XGBoost outperformed LASSO logistic regression for all tasks. Other performance metrics including AUC, balanced accuracy, and accuracy are shown in Figures S9–S16. Figure S17 shows a comparison of F1-Scores of the PA intensity recognition task using LOO and LPO for the LPP and HPP groups, respectively. Similar patterns seen in the PA type recognition task can be found here.

**Figure 4:**
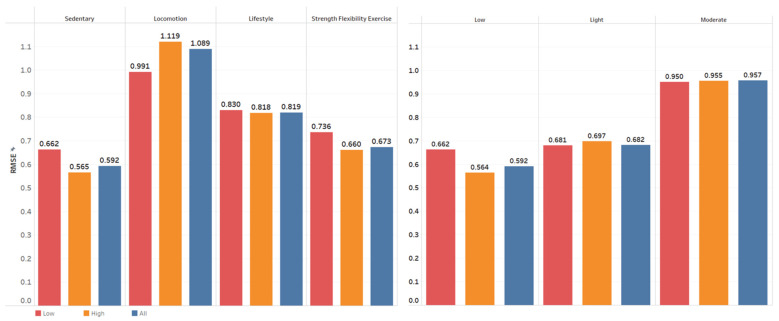
The breakdown of RMSE in energy expenditure estimation task for each activity type and intensity using XGBoost. Low, high, and all groups represent models built for low physical performance group, high physical performance group, and all cohort, respectively.

Across activity type and intensity categories, locomotion and moderate-intensity activities had the lowest performance (highest RMSE) for both the LPP and HPP groups. Sedentary and low-intensity activities had the best (lowest RMSE) for both the LPP and HPP groups, as shown in Figure 4. Meanwhile, XGBoost models obtained a slightly better performance (lower RMSE) for the HPP group than the LPP group in EE estimation for all activity types except locomotion activities. Training on one group and testing on the other leads to slightly worse performance (increased RMSE) in estimating energy expenditure, as shown in Figure S20. Combining data from both groups results in similar performance to the models which were trained and tested on the same group. Table 3 shows the performance of recognizing individual PA using XGBoost. Activities mainly involving wrist movements (computer work, washing windows, digging, etc.) perform better than others. In addition, the models’ performance from the HPP group was uniformly higher than the LPP group across all individual activities except for the strength exercise leg curl.

Figures S21 and S22 show the confusion matrices of recognizing PA type for the HPP and LPP groups. It can be seen that the confusion increases as we move from locomotion and sedentary to SFE PA type. The confusion matrices of recognizing PA intensity for the HPP and LPP groups are shown in Figures S23 and S24. The confusion increases as we move from low to moderate, then light PA intensity.

Figures S25–S28 show the top 15 most important features in recognizing PA type for the HPP and LPP groups generated from the XGBoost models. Figures S29–S31 show the top 15 most important features in recognizing PA intensity for the HPP and LPP groups. Within each recognition task, the ranking of features is similar for both groups.

## 4. Discussion

The goal of this study was to examine the robustness of machine learning models used to recognize the hallmark measures of physical activities to the differences in physical function of older adults. The results demonstrate that the ML algorithms were capable of accurately recognizing PA type and intensity categories as well as estimating EE in both low- and high-functioning older adults. However, individual activity recognition performed less optimally and was systematically more accurate in the higher-functioning group. Overall, our hypothesis that lower physical function would negatively impact the accuracy of ML models was not accepted.

The ML models built in this work showed slightly better performance for the HPP group compared to the LPP group. This slight difference is consistent with the SPPB scores. Poor SPPB scores have been shown to be associated with an increased short- and long-term disability risk [41,42], falls, and frailty in older adults [43,44,45]. These limitations can potentially constrain the ability of individuals to perform their daily activities and would all influence the tri-axial accelerometer output. However, ML models were robust to the variations in physical functions and could represent the accelerometry patterns in the data with only slight differences. 

Examining the robustness of the ML models built on one group and tested on the other (e.g., training on the HPP group and testing on the LPP, and vice versa) showed slight differences between training and testing on the data from the same group compared to training and testing on different groups. For example, training on the HPP group and testing on the LPP group resulted in slightly better performance than training and testing on the LPP group. This emphasizes that the HPP group has less movement variability compared to the LPP group. This can result in more stable ML models. On the other hand, mixing data from the HPP and LPP groups does not have a negative impact on the models’ performance on the HPP group. This is important for researchers who have already collected data in the past and would like to utilize these models for new data collected with some variation in population characteristics, such as physical performance. Researchers may cautiously consider utilizing previous models or data, despite differences in physical performance. These results are consistent with our work focusing on age differences [12]. 

The METs RMSE of EE estimation of activities with higher metabolic intensity is substantially higher than that of activities with lower metabolic intensity. That is an important message for practitioners. Our findings are consistent with previous literature which estimated EE using depth-camera and inertial sensors [46,47,48]. The performance of models built to recognize individual physical activities was lower than those recognizing PA type and intensity categories. We expected lower performance based on previous literature and our own contributions [12,14]. The cause of poor performance is challenging to completely decipher, but it is likely related to the biomechanical overlap of wrist movements between individual tasks. This overlap is less common when estimating activity type categories, which could contribute to their better performance. Additionally, the HPP group appeared to uniformly perform better than the LPP group, albeit still at relatively low accuracy. This finding could be partially explained by the LPP collectively using more compensatory strategies to complete the task (e.g., holding onto a wall to brace themselves). As a result, the altered biomechanics could confuse ML models to a greater extent than the HPP group. Overall, recognition of individual tasks from the wrist position remains problematic when placed in the context of other tasks with similar maneuvers. 

The scaled impurity-based feature importance ranking generated from the XGBoost algorithm demonstrates how relevant these features are to the problem at hand and aids in deeper understanding of the model. The feature importance ranking is consistent for both physical performance groups within each classification task. For example, statistical features depicting the variability in vector magnitudes such as sdvm and cv_vm were important in recognizing sedentary physical activities. However, summary statistics from the *Y*-axis such as mean_Y and moment_Y_three were more relevant in predicting locomotion activities. Additionally, feature importance rankings for the HPP and LPP groups are consistent, indicating that the features are robust to potential movement differences in older adults due to physical function. By examining the important features for the recognition problem at hand, investigators may enhance the performance of the model with fewer computing resources.

Comparing the results with relevant literature is an intricate endeavor due to the differences in the data collection environment and variables considered in the study. Numerous factors vary in different studies, including: number of participants and their characteristics, devices used for data collection, their configuration and placement, number and types of physical activities performed, data collection settings (lab versus free-living), data preprocessing such as the length of the window size to segment the serial accelerometer data and statistical features extracted, etc. Nevertheless, essential comparisons can be made. For example, Ellis et al. [49] built random forest classifier and regression trees on accelerometer data collected from the non-dominant wrist to predict the PA type and estimate PA energy expenditure. They trained and validated their models on forty adults with an average age of 35.8 to predict four activity types (household, stairs, walking, and running). They achieved an average accuracy of 87.5% in predicting the four activity types and 80.2% in predicting eight individual activities. Additionally, they obtained a per minute RMSE value of 1.00 METs, which is slightly higher than (not as good as) the METs we obtained for both the HPP and LPP groups. Davoudi et al. [14] applied four machine learning models, including random forest, support vector machines, neural networks, and decision trees to recognize PA intensity (sedentary, light, and moderate), type (sedentary and locomotion) and individual PAs, and estimate energy expenditure. Their data were collected on 40 participants (an average age of 55.2 years) who performed 15 daily activities. The random forest model obtained an overall accuracy of 87% on PA intensity which is slightly higher than the performance of the models built on the LPP group, but comparable to the accuracy of the HPP group. They obtained an RMSE value of 0.71 METs, an accuracy of 98% on recognizing sedentary activities, and an accuracy of 100% on recognizing locomotion activities, which are slightly better than the HPP group. Compared to other studies that used machine learning techniques, the results from the current work are either slightly better or comparable for both the HPP and LPP groups. Despite the different study settings listed above, we provided a summary of how our work compares with others who utilized machine learning to recognize PA type, intensity, and individual activities or estimate energy expenditure with data from a single triaxial accelerometer placed on the wrist, as shown in Supplementary Table S1. 

The present study had two limitations that need to be acknowledged. First, the data were collected in controlled laboratory settings, which may not represent the activity patterns of the older adults in free-living conditions. However, validating ML models on data collected in controlled laboratory settings is an appropriate first step in evaluating PA recognition methods [50]. Collecting data in free-living conditions can better reflect the activity patterns and transitions among activities, but this is challenged by the need to label data. Second, the window size applied in the current study may not be optimal for all tasks and physical performance groups. Future work can focus on evaluating window sizes for improving models’ performance. 

## 5. Conclusions

Machine learning models can accurately recognize PA types and intensity categories and estimate energy expenditure in older adults with high and low physical function. Estimation of individual activities requires additional work. In summary, processing wrist-worn accelerometer data collected from older adults without considering individual physical performance is sufficient for estimating the hallmark measures of physical activities. Researchers can cautiously consider utilizing previously collected data and models, despite the well-known age-related variability in physical function.

## Figures and Tables

**Table 1:** Description of features extracted from the raw data.

	Feature	Description
**Time**	Mean of vector magnitude and acceleration from 3 axes (mvm, mean_x, mean_y, and mean_z)	Sample mean of VM, acceleration from *x*-, *y*-, and *z*-axis in the window
	SD of vector magnitude and acceleration from 3 axes (sdvm, sd_x, sd_y, and sd_z)	Sample standard deviation of VM, acceleration from *x*-, *y*-, and *z*-axis in the window
	Coefficient of variation of vector magnitude and acceleration from 3 axes (cv_vm, cv_x, cv_y, and cv_z)	Standard deviation of VM, acceleration from *x*-, *y*-, and *z*-axis in the window divided by the mean, multiplied by 100
	The minimum value of vector magnitude and acceleration from 3 axes (min_vm, min_x, min_y, and min_z)	The minimum value of VM and acceleration from *x*-, *y*-, and *z*-axis in the window
	The maximum value of vector magnitude (max_vm, max_x, max_y, and max_z)	The maximum value of VM and acceleration from *x*-, *y*-, and *z*-axis in the window
	25% quantile of vector magnitude and acceleration from 3 axes (lower_vm_25, lower_x_25, lower_y_25, and lower_z_25)	Lower 25% quantile of VM and acceleration from *x*-, *y*-, and *z*-axis in the window
	75% quantile of vector magnitude and acceleration from 3 axes (upper_vm_75, upper_x_75, upper_y_75, and upper_z_75)	Upper 75% quantile of VM and acceleration from *x*-, *y*-, and *z*-axis in the window
	Third moment of vector magnitude and acceleration from 3 axes (third_vm, third_x, third_y, and third_z)	Third moment of VM and acceleration from *x*-, *y*-, and *z*-axis in the window
	Fourth moment of vector magnitude and acceleration from 3 axes (fourth_vm, fourth_x, fourth_y, and fourth_z)	Fourth moment of VM and acceleration from *x*-, *y*-, and *z*-axis in the window
	Skewness of vector magnitude and acceleration from 3 axes (skewness_vm, skewness_x, skewness_y, and skewness_z)	Skewness of VM, acceleration from *x*-, *y*-, and *z*-axis in the window
	Kurtosis of vector magnitude and acceleration from 3 axes (kurtosis_vm, kurtosis_x, kurtosis_y, and kurtosis_z)	Kurtosis of VM, acceleration from *x*-, *y*-, and *z*-axis in the window
	Mean angle of acceleration relative to vertical on the device (mangle)	Sample mean of the angle between *x*-axis and VM in the window
	SD of the angle of acceleration relative to vertical on the device (sdangle)	Sample standard deviation of the angles in the window
**Frequency**	Percentage of the power of the vm that is in 0.6–2.5 Hz (p625)	Fraction of power within human movement frequencies (i.e., 0.6–2.5 Hz)
	Dominant frequency of vm (df)	Frequency corresponding to the largest modulus
	Fraction of power in vm at the dominant frequency (fpdf)	Modulus of the dominant frequency/sum of moduli at each frequency

**Table 2:** Descriptive characteristics of participants by physical performance group.

		Low	High	All
**Age Range, years**		[62–89]	[60–88]	[60–89]
**Mean Age (SD), years**		75.9 (6.6)	70.3 (6.6)	72.4 (7.1)
**Mean SPPB Scores (SD)**		7.7 (1.8)	11.3 (0.8)	10.0 (2.1)
**Mean Walk Speed (SD), m/s**		0.79 (0.15)	1.05 (0.17)	0.95 (0.20)
**Mean BMI (SD), kg/m^2^**		30.8 (8.8)	27.5 (4.8)	28.7 (6.7)
**Women %**		56.9%	57.2%	57.1%
**Race**	White	77.0%	89.1%	84.6%
	Black	8.8%	5.1%	6.5%
	Asian	3.3%	1.9%	2.4%
	American Indian or Alaska Native	2.2%	1.9%	2.0%
	Other	0%	1.9%	1.2%
	Refuse	2.2%	1.3%	1.6%
**Chronic Health Conditions**	High Blood Pressure %	59.3%	41.7%	48.2%
	Congestive Heart Failure %	3.3%	1.9%	2.4%
	Stroke %	4.4%	3.2%	3.6%
	Diabetes %	22.0%	12.8%	16.2%
	Hypothyroidism %	14.3%	14.1%	14.2%
	Chronic Lung Disease %	23.1%	9.6%	14.6%
	Chronic Heart Disease %	15.4%	6.4%	9.7%
	Chronic Liver Disease %	7.7%	3.2%	4.9%
	Chronic Kidney Disease %	23.1%	12.2%	16.2%
	Osteoporosis %	13.2%	13.5%	13.4%
	Mean Disease Index (SD)	1.86 (1.49)	1.19 (1.14)	1.43 (1.32)
**Total number of participants**		91	156	247

**Table 3:** The F1-Scores of individual physical activity recognition task using XGBoost. Each value is the mean and standard deviation of the five-fold nested cross-validation.

Individual Activities Recognition Performance (F1-Score)						
	Activity Type Category	Activity Intensity Category	Low	High	Absolute Difference	All
COMPUTER WORK (*n* = 66, 130, 196)	Sedentary	Low	0.72 (0.05)	0.78 (0.03)	0.06	0.78 (0.01)
TV WATCHING (*n* = 69, 133, 202)	Sedentary	Low	0.57 (0.03)	0.67 (0.03)	0.1	0.65 (0.04)
STANDING STILL (*n* = 70, 132, 202)	Sedentary	Low	0.44 (0.07)	0.65 (0.03)	0.21	0.60 (0.05)
STAIR DESCENT (*n* = 60, 130, 190)	Locomotion	Moderate	0.61 (0.09)	0.68 (0.03)	0.07	0.68 (0.03)
STAIR ASCENT (*n* = 41, 118, 159)	Locomotion	Moderate	0.43 (0.07)	0.57 (0.05)	0.14	0.58 (0.03)
RAPID WALK (*n* = 75, 138, 213)	Locomotion	Moderate	0.40 (0.11)	0.54 (0.03)	0.13	0.52 (0.04)
LEISURE WALK (*n* = 79, 138, 217)	Locomotion	Moderate	0.44 (0.05)	0.52 (0.03)	0.08	0.48 (0.02)
WALKING AT RPE 5 (*n* = 59, 135, 194)	Locomotion	Moderate	0.40 (0.07)	0.42 (0.06)	0.02	0.44 (0.03)
WALKING AT RPE 1 (*n* = 71, 134, 205)	Locomotion	Moderate	0.32 (0.04)	0.44 (0.05)	0.12	0.42 (0.02)
STRENGTH EXERCISE CHEST PRESS (*n* = 62, 117, 179)	SFE	Light	0.57 (0.11)	0.68 (0.06)	0.11	0.68 (0.04)
STRENGTH EXERCISE LEG CURL (*n* = 61, 127, 188)	SFE	Light	0.63 (0.10)	0.62 (0.04)	0.01	0.66 (0.02)
STRETCHING YOGA (*n* = 67, 131, 198)	SFE	Light	0.50 (0.04)	0.65 (0.02)	0.15	0.61 (0.04)
STRENGTH EXERCISE LEG EXTENSION (*n* = 57, 125, 182)	SFE	Light	0.21 (0.03)	0.41 (0.05)	0.2	0.39 (0.05)
WASHING WINDOWS (*n* = 65, 130, 195)	Life-Style	Moderate	0.66 (0.04)	0.76 (0.04)	0.1	0.75 (0.04)
DIGGING (*n* = 63, 133, 196)	Life-Style	Moderate	0.56 (0.06)	0.72 (0.05)	0.16	0.69 (0.04)
IRONING (*n* = 64, 128, 192)	Life-Style	Light	0.56 (0.06)	0.69 (0.03)	0.13	0.68 (0.01)
MOPPING (*n* = 64, 126, 190)	Life-Style	Moderate	0.59 (0.04)	0.70 (0.03)	0.11	0.68 (0.04)
WASHING DISHES (*n* = 71, 136, 207)	Life-Style	Light	0.57 (0.07)	0.68 (0.02)	0.1	0.67 (0.03)
REPLACING SHEETS ON A BED (*n* = 67, 130, 197)	Life-Style	Moderate	0.55 (0.05)	0.64 (0.02)	0.09	0.65 (0.02)
HEAVY LIFTING (*n* = 52, 125, 177)	Life-Style	Moderate	0.42 (0.08)	0.65 (0.04)	0.23	0.63 (0.03)
PERSONAL CARE (*n* = 71, 135, 206)	Life-Style	Light	0.54 (0.03)	0.67 (0.03)	0.13	0.63 (0.01)
UNLOADING STORING DISHES (*n* = 72, 135, 207)	Life-Style	Light	0.59 (0.08)	0.63 (0.02)	0.04	0.63 (0.03)
SWEEPING (*n* = 69, 136, 205)	Life-Style	Moderate	0.42 (0.05)	0.62 (0.01)	0.20	0.59 (0.02)
VACUUMING (*n* = 66, 132, 198)	Life-Style	Moderate	0.53 (0.03)	0.62 (0.04)	0.09	0.59 (0.01)
LIGHT GARDENING (*n* = 72, 139, 211)	Life-Style	Moderate	0.45 (0.04)	0.59 (0.02)	0.14	0.58 (0.02)
SHOPPING (*n* = 68, 130, 198)	Life-Style	Light	0.41 (0.06)	0.54 (0.04)	0.13	0.53 (0.01)
LIGHT HOME MAINTENANCE (*n* = 67, 130, 197)	Life-Style	Moderate	0.35 (0.04)	0.56 (0.03)	0.21	0.52 (0.01)
PREPARE SERVE MEAL (*n* = 71, 136, 207)	Life-Style	Light	0.51 (0.04)	0.51 (0.04)	0.00	0.52 (0.02)
LAUNDRY WASHING (*n* = 68, 128, 196)	Life-Style	Light	0.35 (0.07)	0.50 (0.04)	0.15	0.50 (0.02)
YARD WORK (*n* = 69, 130, 199)	Life-Style	Moderate	0.34 (0.03)	0.48 (0.05)	0.14	0.46 (0.03)
STRAIGHTENING UP DUSTING (*n* = 71, 135, 206)	Life-Style	Moderate	0.37 (0.07)	0.43 (0.05)	0.06	0.45 (0.03)
TRASH REMOVAL (*n* = 65, 130, 195)	Life-Style	Moderate	0.28 (0.06)	0.47 (0.04)	0.19	0.44 (0.02)
DRESSING (*n* = 65, 130, 195)	Life-Style	Light	0.33 (0.04)	0.45 (0.03)	0.12	0.44 (0.02)

* RPE: Rate of Perceived Exertion (0–10).

## Supplementary Materials

The following supporting information can be downloaded at: https://www.mdpi.com/article/10.3390/s22083061/s1, Figure S1: The F1-score of physical activity (PA) type recognition task using LASSO regression. Each value is the mean and standard deviation of the five-fold nested cross validation. Low, high, and all groups represent models built for low physical performance group, high physical performance group, and all cohort, respectively. Figure S2: The receiver operating characteristic-area under the curve of PA type recognition task using XGBoost. Each value is the mean and standard deviation of the five-fold nested cross validation. Low, high, and all groups represent models built for low physical performance group, high physical performance group, and all cohort, respectively. Figure S3: The receiver operating characteristic-area under the curve of PA type recognition task using LASSO regression. Each value is the mean and standard deviation of the five-fold nested cross validation. Low, high, and all groups represent models built for low physical performance group, high physical performance group, and all cohort respectively. Figure S4: The accuracy of PA type recognition task using XGBoost. Each value is the mean and standard deviation of the five-fold nested cross validation. Low, high, and all groups represent models built for low physical performance group, high physical performance group, and all cohort, respectively. Figure S5: The accuracy of PA type recognition task using LASSO regression. Each value is the mean and standard deviation of the five-fold nested cross validation. Figure S6: The balanced accuracy of PA type recognition task using XGBoost. Each value is the mean and standard deviation of the five-fold nested cross validation. Low, high, and all groups represent models built for low physical performance group, high physical performance group, and all cohort, respectively. Figure S7: The balanced accuracy of PA type recognition task using LASSO regression. Each value is the mean and standard deviation of the five-fold nested cross validation. Low, high, and all groups represent models built for low physical performance group, high physical performance group, and all cohort, respectively. Figure S8: The comparison of F1-Scores of physical activity type recognition task evaluated by nested cross-validation, LOO and LPO for the LPP and HPP groups. Figure S9: The F1-score of PA intensity recognition task using XGBoost. Each value is the mean and standard deviation of the five-fold nested cross validation. Low, high, and all groups represent models built for low physical performance group, high physical performance group, and all cohort, respectively. Figure S10: The F1-score of PA intensity recognition task using LASSO regression. Each value is the mean and standard deviation of the five-fold nested cross validation. Low, high, and all groups represent models built for low physical performance group, high physical performance group, and all cohort, respectively. Figure S11: The receiver operating characteristic-area under the curve of PA intensity recognition task using XGBoost. Each value is the mean and standard deviation of the five-fold nested cross validation. Low, high, and all groups represent models built for low physical performance group, high physical performance group, and all cohort, respectively. Figure S12: The receiver operating characteristic-area under the curve of PA intensity recognition task using LASSO regression. Each value is the mean and standard deviation of the five-fold nested cross validation. Low, high, and all groups represent models built for low physical performance group, high physical performance group, and all cohort, respectively. Figure S13: The accuracy of PA intensity recognition task using XGBoost. Each value is the mean and standard deviation of the five-fold nested cross validation. Low, high, and all groups represent models built for low physical performance group, high physical performance group, and all cohort, respectively. Figure S14: The accuracy of PA intensity recognition task using LASSO regression. Each value is the mean and standard deviation of the five-fold nested cross validation. Low, high, and all groups represent models built for low physical performance group, high physical performance group, and all cohort, respectively. Figure S15: The balanced accuracy of PA intensity recognition task using XGBoost. Each value is the mean and standard deviation of the five-fold nested cross validation. Low, high, and all groups represent models built for low physical performance group, high physical performance group, and all cohort, respectively. Figure S16: The balanced accuracy of PA intensity recognition task using LASSO regression. Each value is the mean and standard deviation of the five-fold nested cross validation. Low, high, and all groups represent models built for low physical performance group, high physical performance group, and all cohort, respectively. Figure S17: The comparison of F1-Scores of physical activity intensity recognition task evaluated by nested cross-validation, LOO and LPO for the LPP and HPP groups, respectively. Figure S18: The RMSE of energy expenditure estimation task using XGBoost. Each value is the mean and standard deviation of five-fold nested cross-validation. Low, high, and all groups represent models built for low physical performance group, high physical performance group, and all cohort, respectively. Figure S19: The RMSE of energy expenditure estimation task using LASSO regression. Each value is the mean and standard deviation of five-fold nested cross-validation. Low, high, and all groups represent models built for low physical performance group, high physical performance group, and all cohort, respectively. Figure S20: The comparison of RMSE of EE estimation task evaluated by nested cross-validation, LOO and LPO. Figure S21: Confusion matrix of recognizing PA type for low-functioning group. Figure S22: Confusion matrix of recognizing PA type for high-functioning group. Figure S23: Confusion matrix of recognizing PA intensity for low-functioning group. Figure S24: Confusion matrix of recognizing PA intensity for high-functioning group. Figure S25: Feature importance for recognizing sedentary activities for different functioning groups. Figure S26: Feature importance for recognizing locomotion activities for different functioning groups. Figure S27: Feature importance for recognizing life-style activities for different functioning groups. Figure S28: Feature importance for recognizing strength flexibility exercise activities for different functioning groups. Figure S29: Feature importance for recognizing low intensity activities for different functioning groups. Figure S30: Feature importance for recognizing light intensity activities for different functioning groups. Figure S31: Feature importance for recognizing moderate intensity activities for different functioning groups. Table S1: Comparison with relevant work in the literature. The listed studies collected from the wrist position for physical activity type, physical activity intensity and individual activity recognition. classification performance is accuracy unless otherwise stated. For the purpose of comparison, we calculated the average performance for the recognition task (i.e., physical activity type recognition). N is the number of participants; Num is number; ML is machine learning; RMSE is the root mean square error; RF is random forest; SVM is support vector machine; DT is decision tree and ANN is artificial neural network.
