# Supplementary material for: Are Machine Learning Models on Wrist Accelerometry Robust against Differences in Physical Performance among Older Adults?
Source: Sensors (Basel). 2022 Apr 15;22(8):3061. doi: 10.3390/s22083061 (PMC9032589; doi:10.3390/s22083061)
Supplement: Supplementary file 1 [file sensors-22-03061-s001.zip › sensors-1596731-supplementary.pdf]

sensors

MDPI

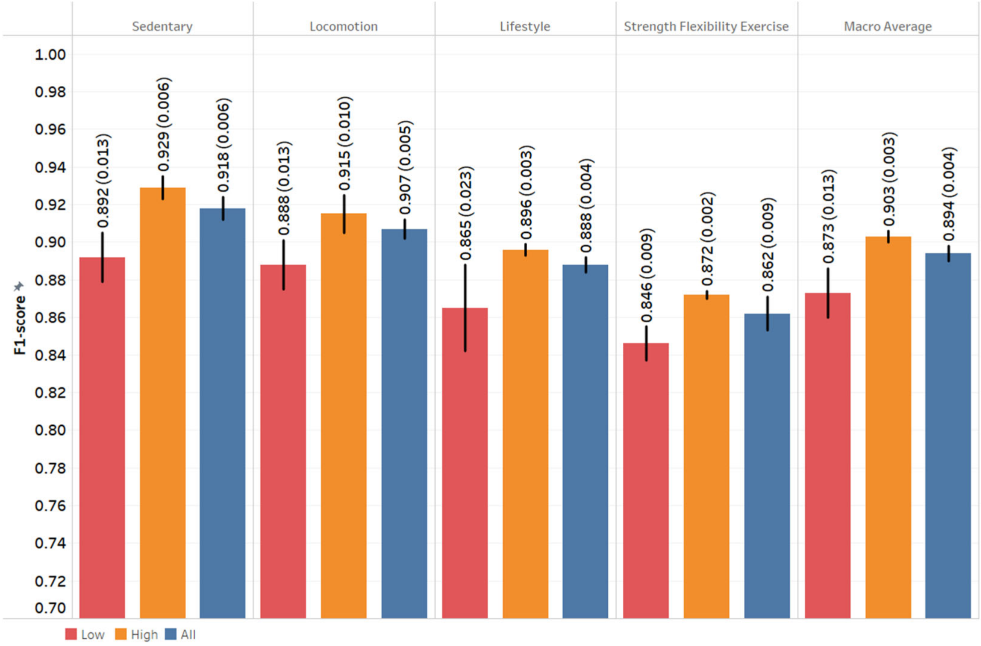

Figure S1. The F1-score of physical activity (PA) type recognition task using LASSO regression. Each value is the mean and standard deviation of the 5-fold nested cross validation. Low, high, and all groups represent models built for low physical performance group, high physical performance group, and all cohort respectively.

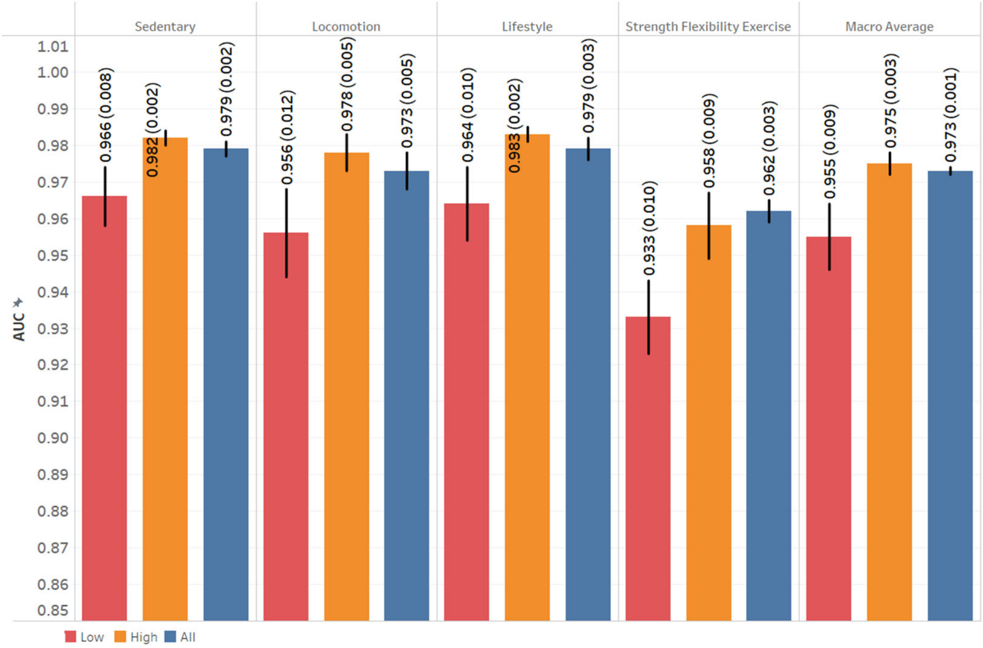

Figure S2. The receiver operating characteristic-area under the curve of PA type recognition task using XGBoost. Each value is the mean and standard deviation of the 5-fold nested cross validation. Low, high, and all groups represent models built for low physical performance group, high physical performance group, and all cohort respectively.

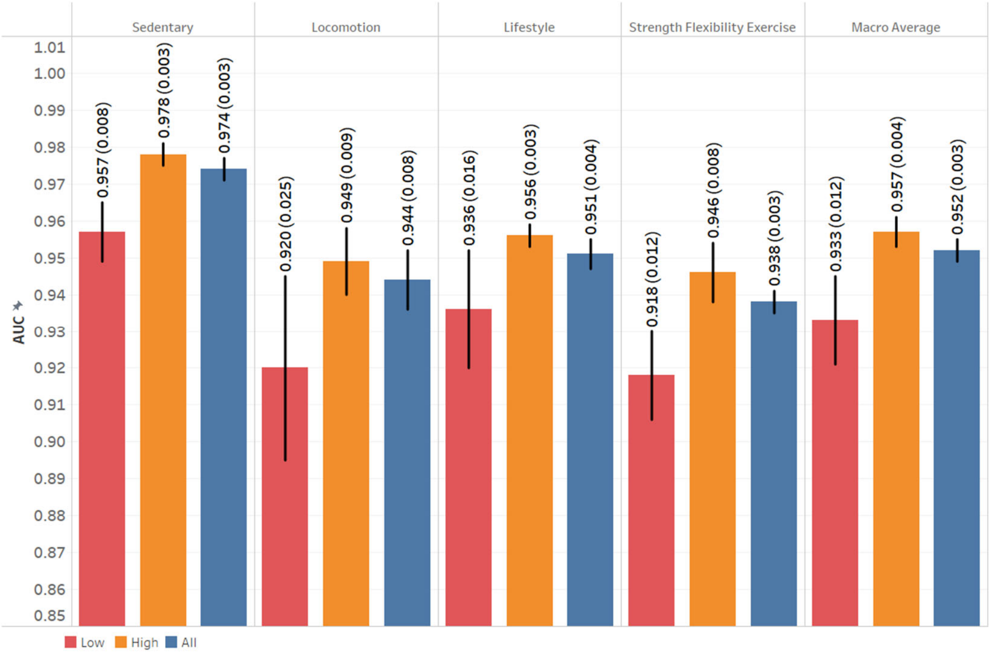

Figure S3. The receiver operating characteristic-area under the curve of PA type recognition task using LASSO regression. Each value is the mean and standard deviation of the 5-fold nested cross validation. Low, high, and all groups represent models built for low physical performance group, high physical performance group, and all cohort respectively.

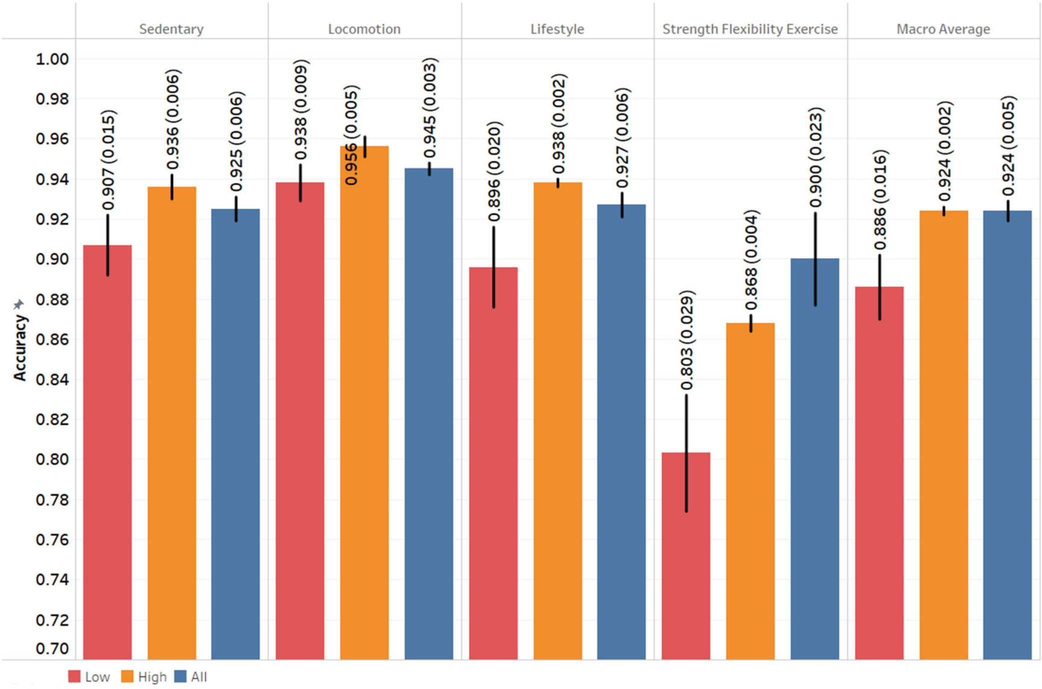

Figure S4. The accuracy of PA type recognition task using XGBoost. Each value is the mean and standard deviation of the 5-fold nested cross validation. Low, high, and all groups represent models built for low physical performance group, high physical performance group, and all cohort respectively.

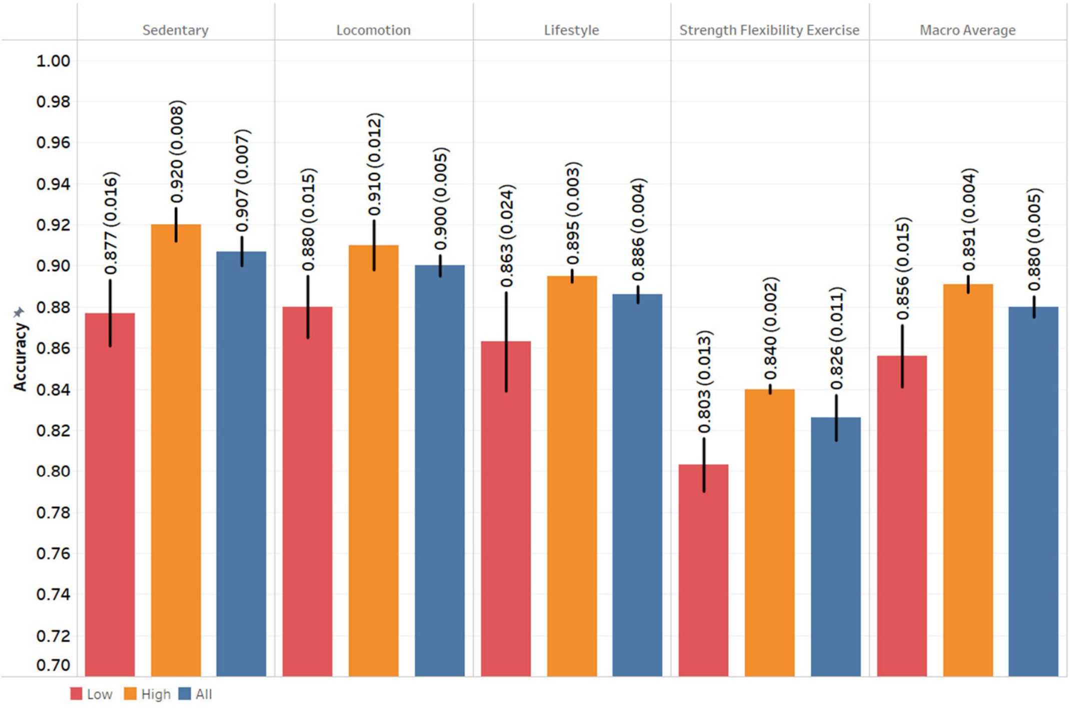

Figure S5. The accuracy of PA type recognition task using LASSO regression. Each value is the mean and standard deviation of the 5-fold nested cross validation.

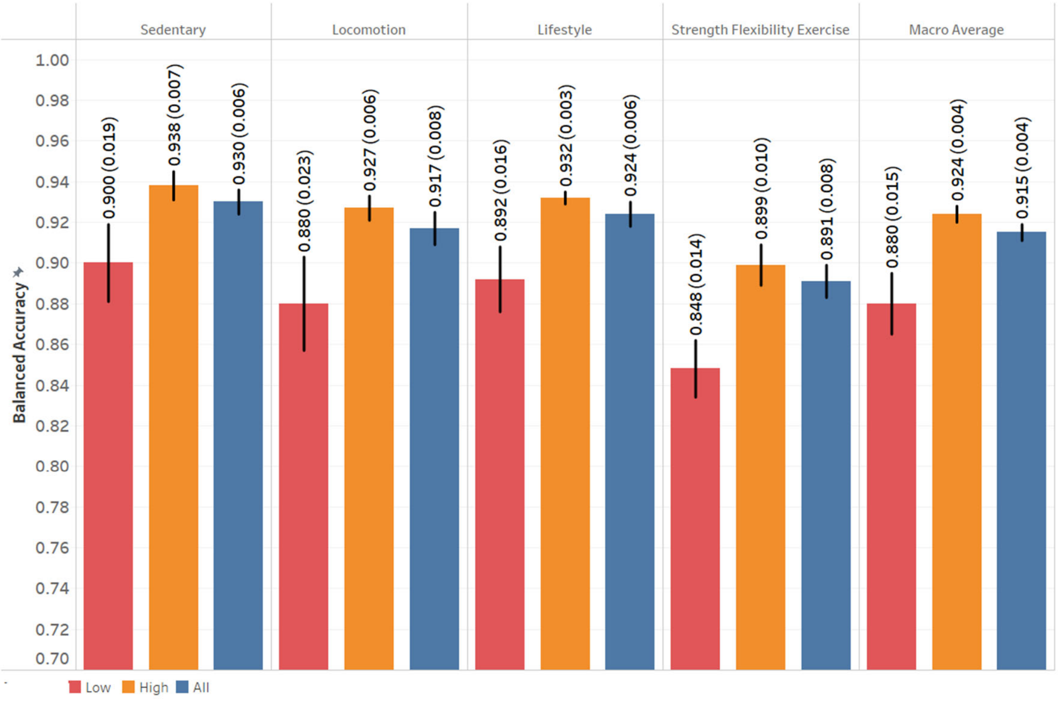

Figure S6. The balanced accuracy of PA type recognition task using XGBoost. Each value is the mean and standard deviation of the 5-fold nested cross validation. Low, high, and all groups represent models built for low physical performance group, high physical performance group, and all cohort respectively.

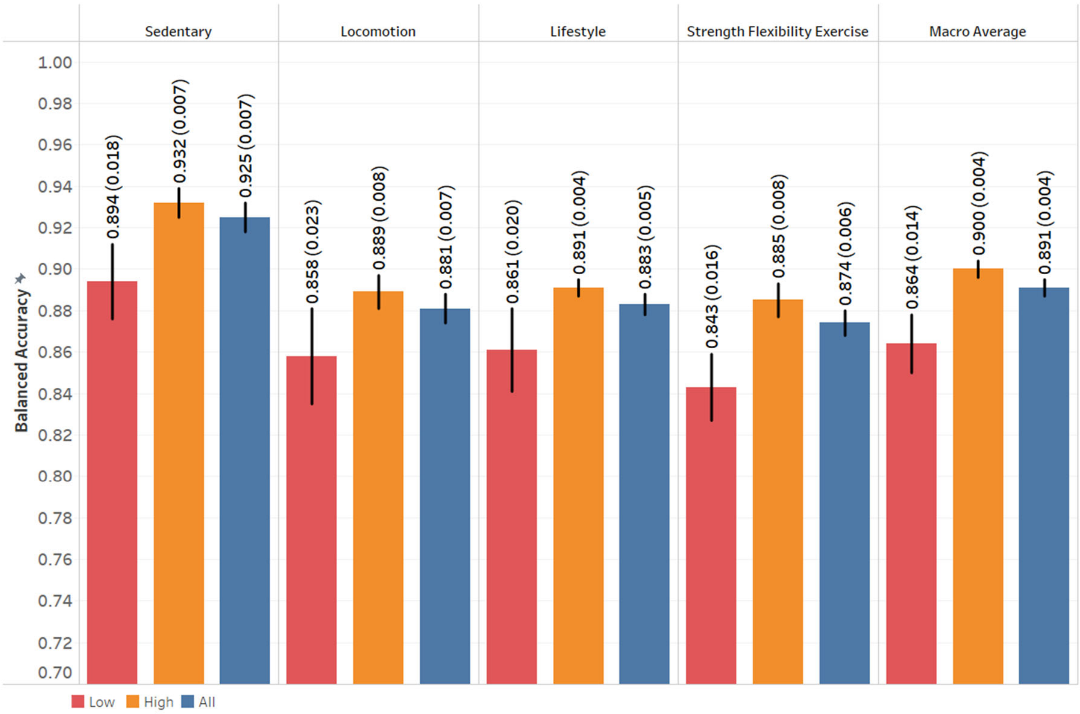

Figure S7. The balanced accuracy of PA type recognition task using LASSO regression. Each value is the mean and standard deviation of the 5-fold nested cross validation. Low, high, and all groups represent models built for low physical performance group, high physical performance group, and all cohort respectively.

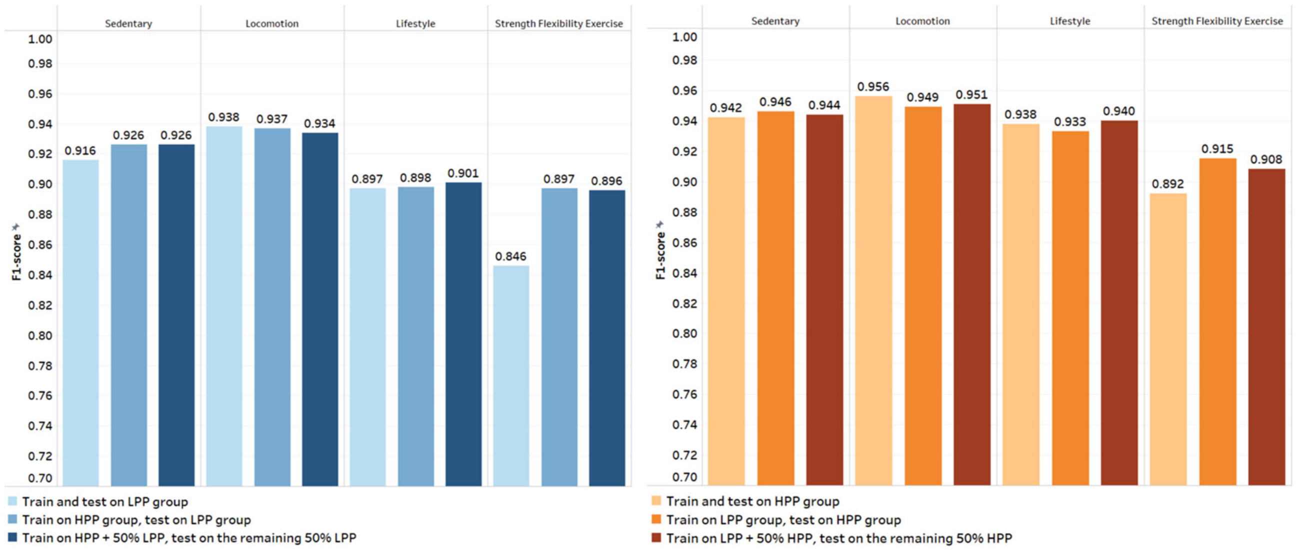

Figure S8. The comparison of F1-Scores of physical activity type recognition task evaluated by nested cross-validation, LOO and LPO for the LPP and HPP groups.

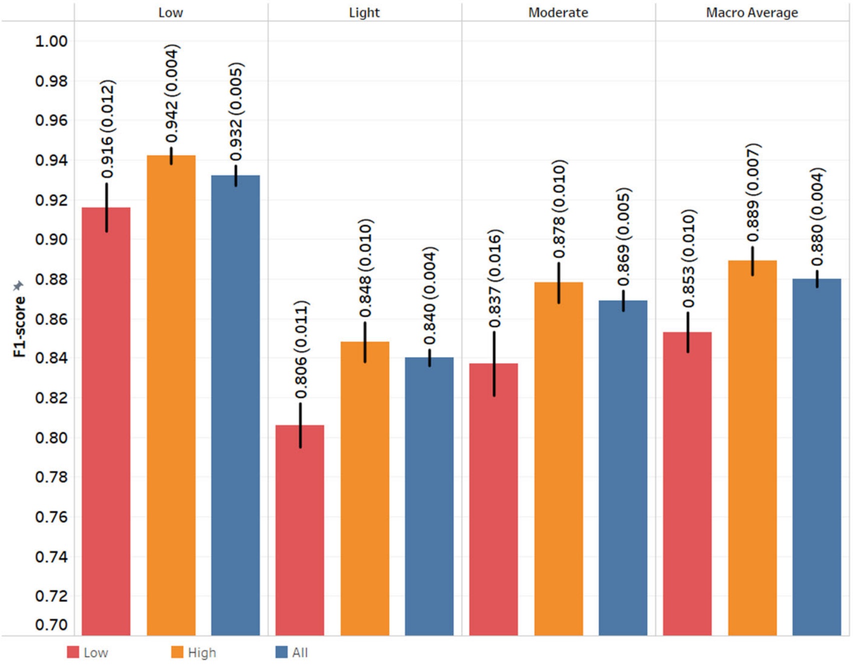

Figure S9. The F1-score of PA intensity recognition task using XGBoost. Each value is the mean and standard deviation of the 5-fold nested cross validation. Low, high, and all groups represent models built for low physical performance group, high physical performance group, and all cohort respectively.

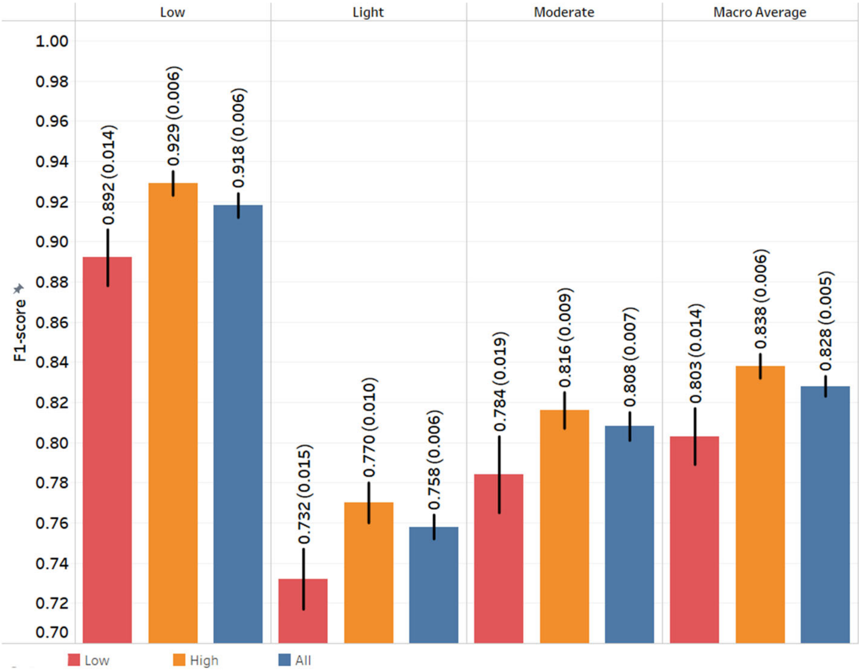

Figure S10. The F1-score of PA intensity recognition task using LASSO regression. Each value is the mean and standard deviation of the 5-fold nested cross validation. Low, high, and all groups represent models built for low physical performance group, high physical performance group, and all cohort respectively.

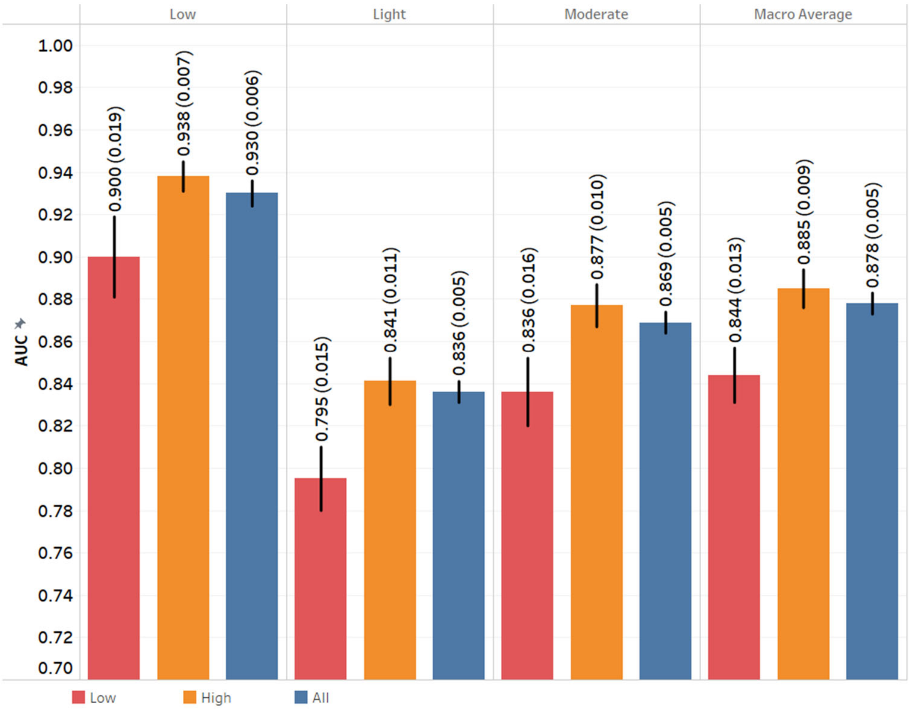

Figure S11. The receiver operating characteristic-area under the curve of PA intensity recognition task using XGBoost. Each value is the mean and standard deviation of the 5-fold nested cross validation. Low, high, and all groups represent models built for low physical performance group, high physical performance group, and all cohort respectively.

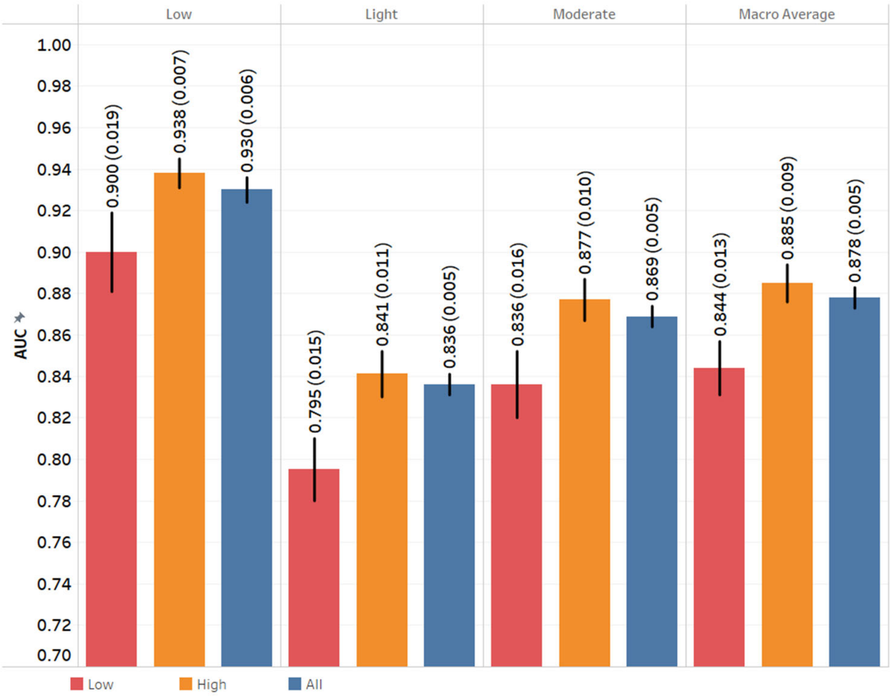

Figure S12. The receiver operating characteristic-area under the curve of PA intensity recognition task using LASSO regression. Each value is the mean and standard deviation of the 5-fold nested

cross validation. Low, high, and all groups represent models built for low physical performance group, high physical performance group, and all cohort respectively.

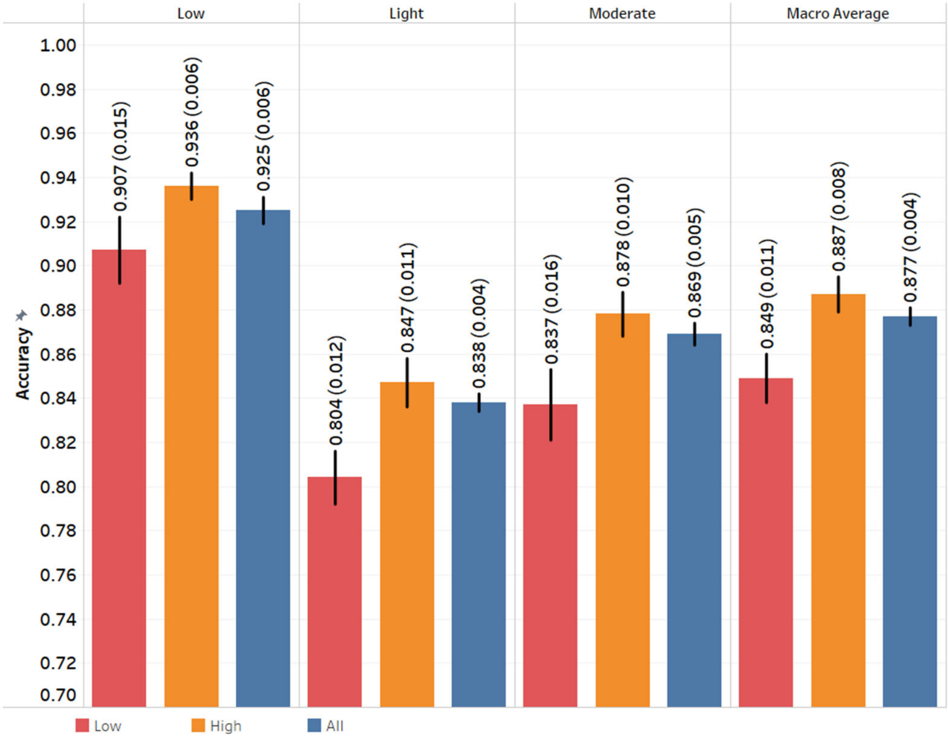

Figure S13. The accuracy of PA intensity recognition task using XGBoost. Each value is the mean and standard deviation of the 5-fold nested cross validation. Low, high, and all groups represent models built for low physical performance group, high physical performance group, and all cohort respectively.

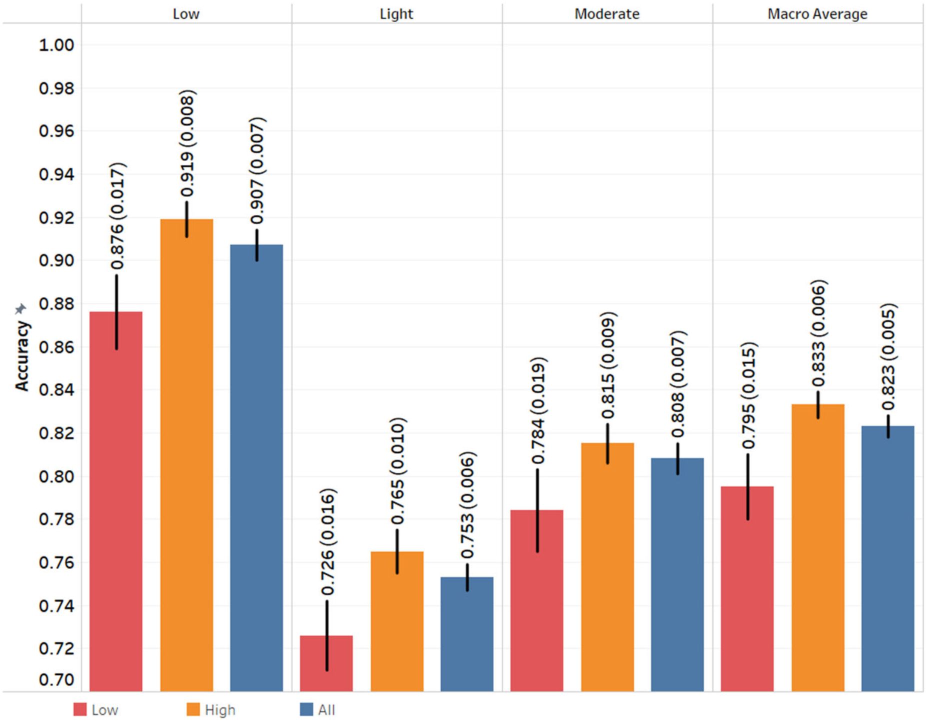

Figure S14. The accuracy of PA intensity recognition task using LASSO regression. Each value is the mean and standard deviation of the 5-fold nested cross validation. Low, high, and all groups represent models built for low physical performance group, high physical performance group, and all cohort respectively.

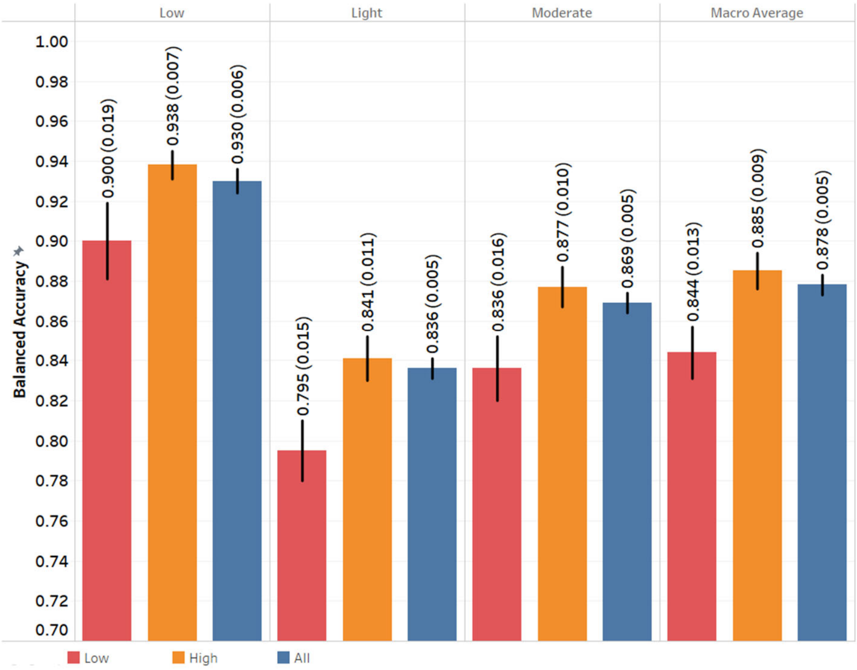

Figure S15. The balanced accuracy of PA intensity recognition task using XGBoost. Each value is the mean and standard deviation of the 5-fold nested cross validation. Low, high, and all groups

represent models built for low physical performance group, high physical performance group, and all cohort respectively.

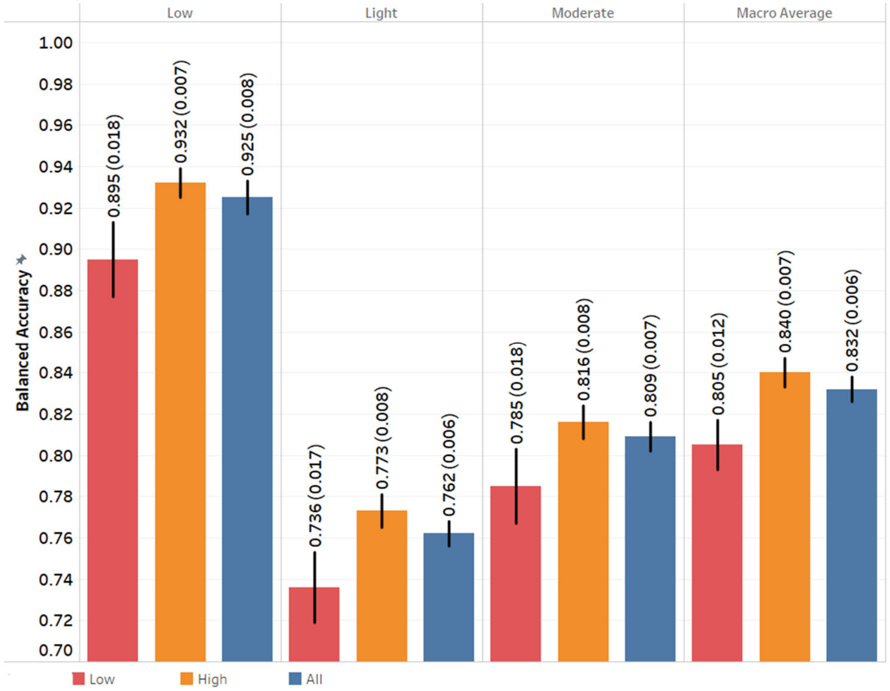

Figure S16. The balanced accuracy of PA intensity recognition task using LASSO regression. Each value is the mean and standard deviation of the 5-fold nested cross validation. Low, high, and all groups represent models built for low physical performance group, high physical performance group, and all cohort respectively.

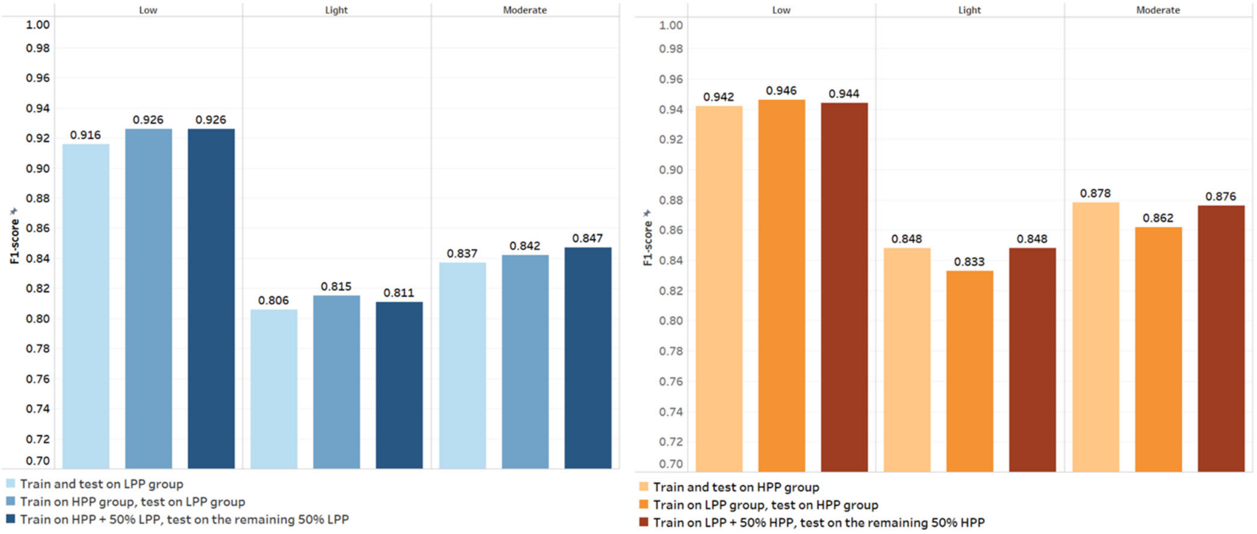

Figure S17. The comparison of F1-Scores of physical activity intensity recognition task evaluated by nested cross-validation, LOO and LPO for the LPP and HPP groups respectively.

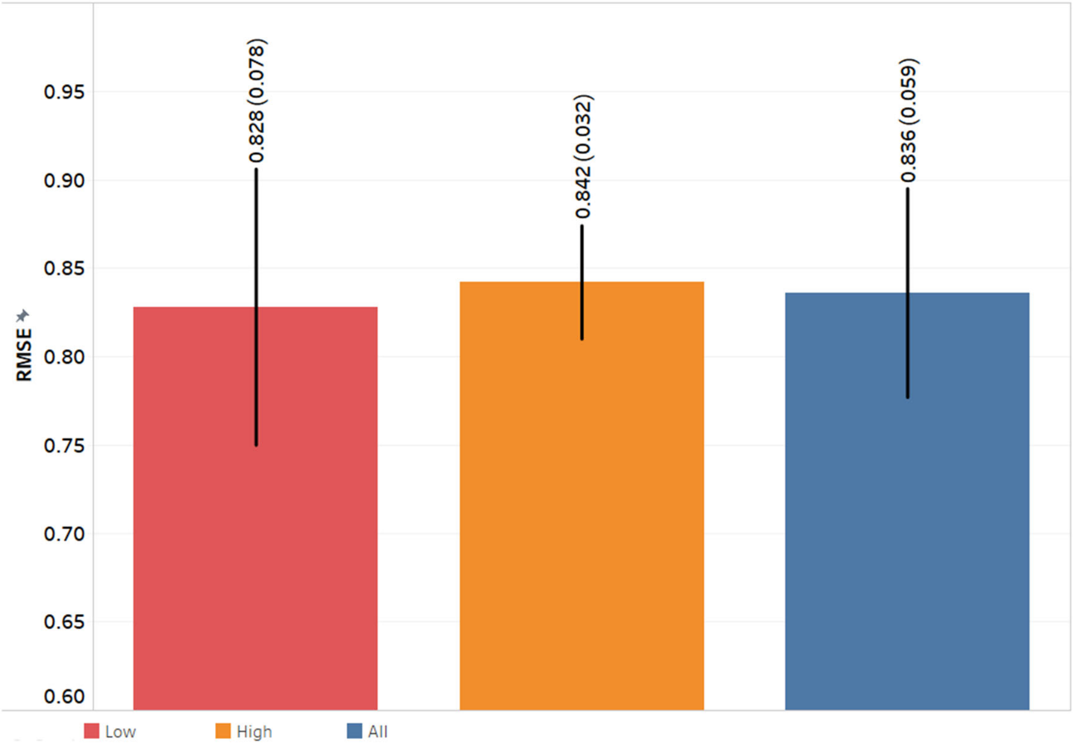

Figure S18. The RMSE of energy expenditure estimation task using XGBoost. Each value is the mean and standard deviation of 5-fold nested cross-validation. Low, high, and all groups represent models built for low physical performance group, high physical performance group, and all cohort respectively.

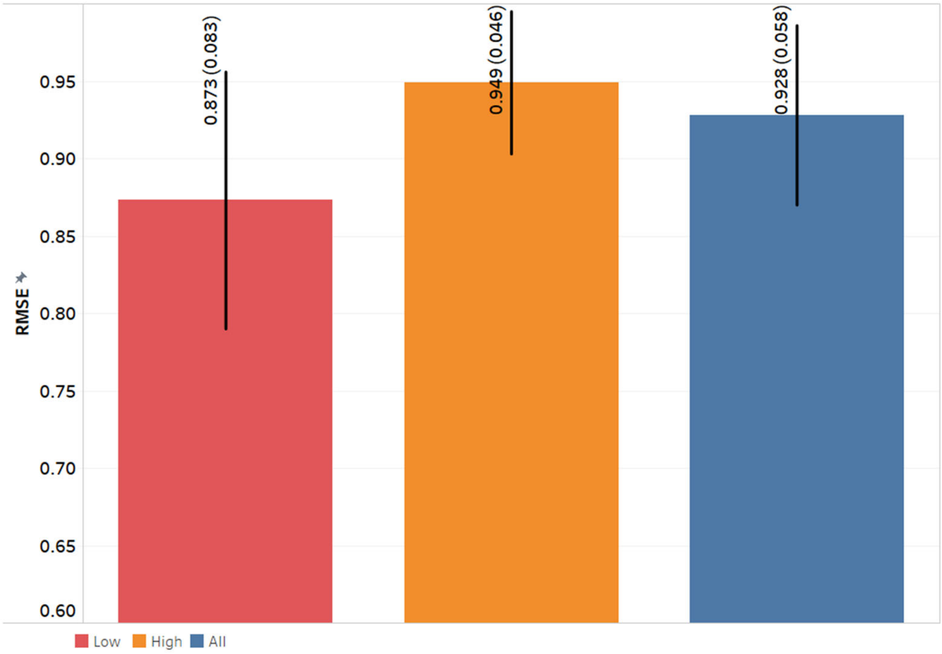

Figure S19. The RMSE of energy expenditure estimation task using LASSO regression. Each value is the mean and standard deviation of 5-fold nested cross-validation. Low, high, and all groups represent models built for low physical performance group, high physical performance group, and all cohort respectively.

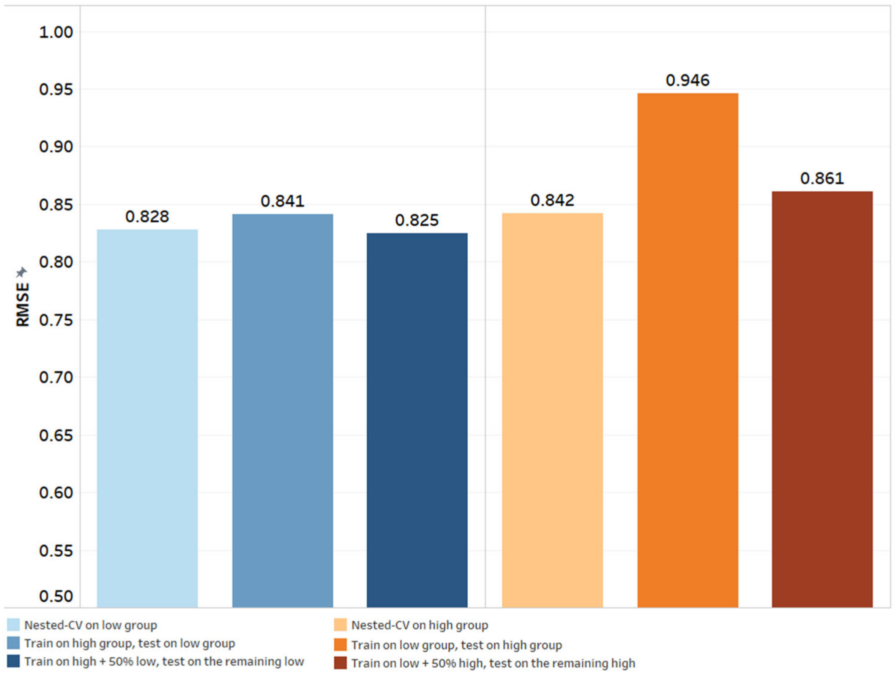

Figure S20. The comparison of RMSE of EE estimation task evaluated by nested cross-validation, LOO and LPO.

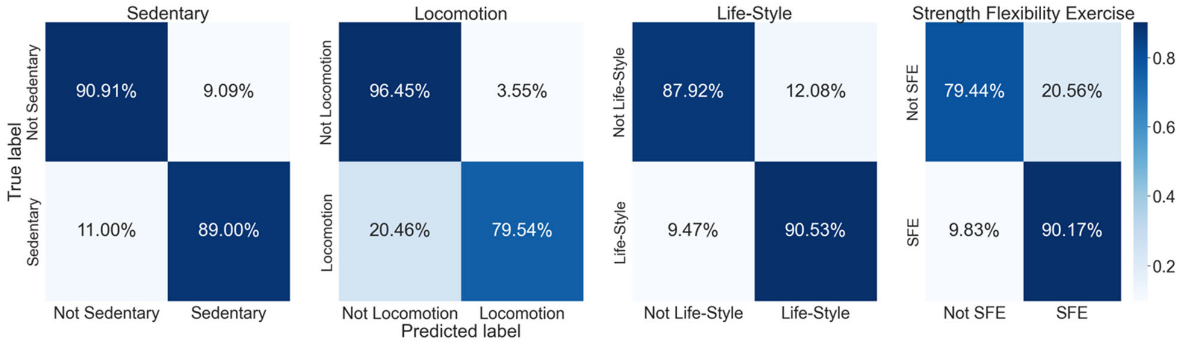

Figure S21. Confusion matrix of recognizing PA type for low functioning group.

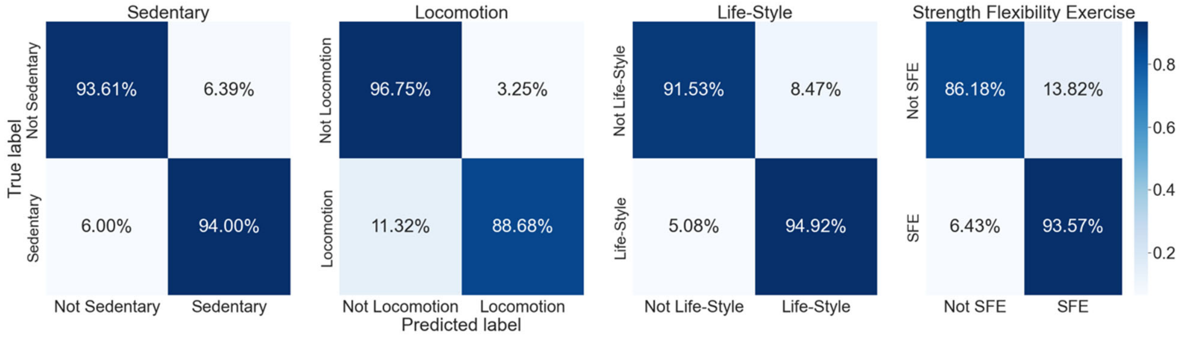

Figure S22. Confusion matrix of recognizing PA type for high functioning group.

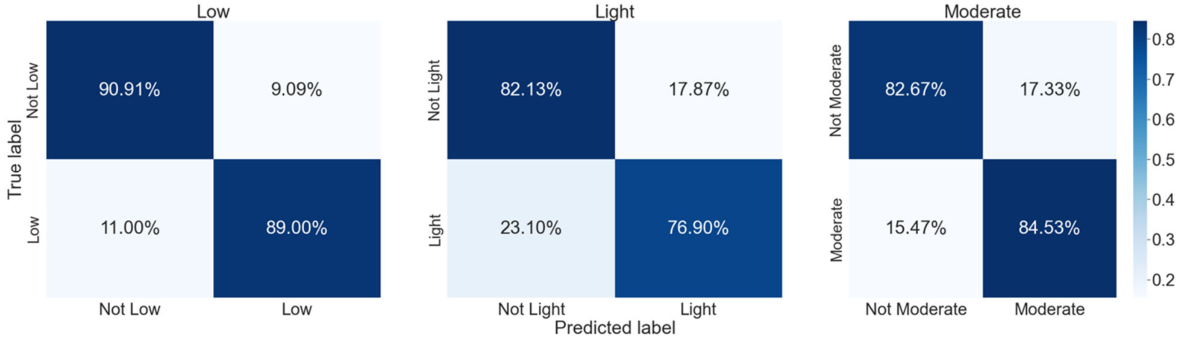

Figure S23. Confusion matrix of recognizing PA intensity for low functioning group.

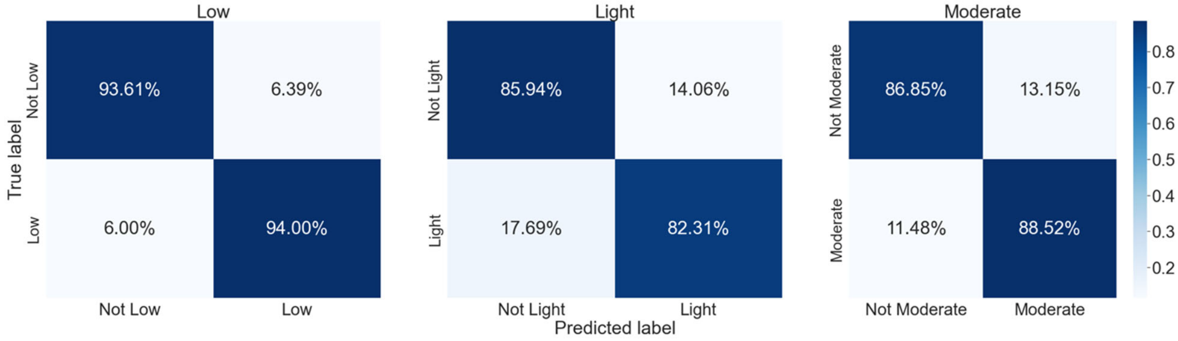

Figure S24. Confusion matrix of recognizing PA intensity for high functioning group.

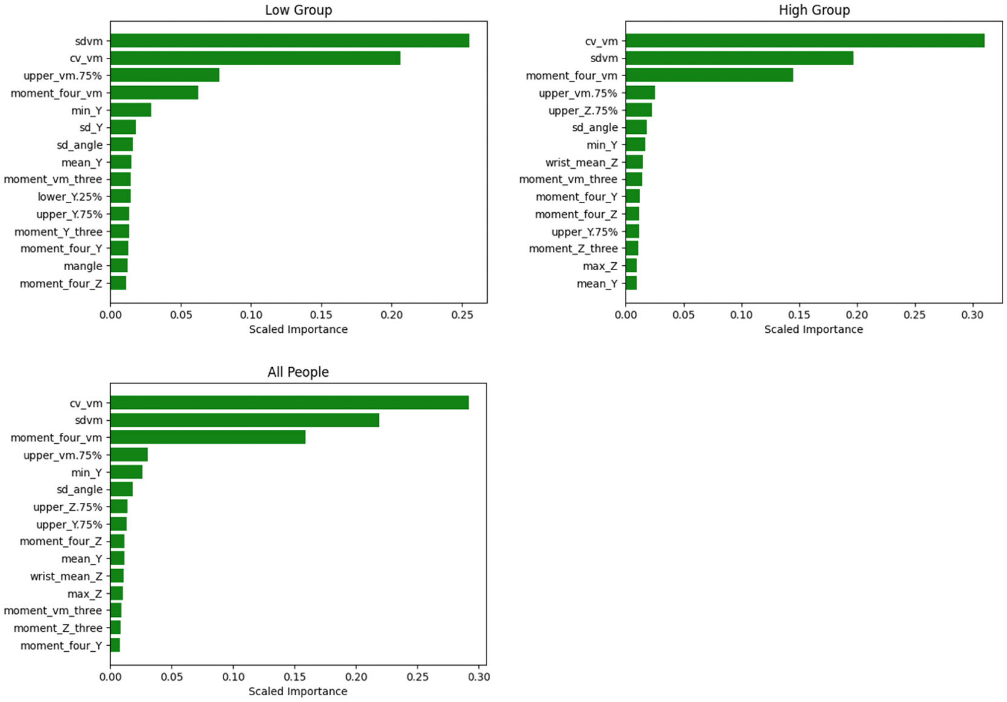

Figure S25. Feature importance for recognizing sedentary activities for different functioning groups.

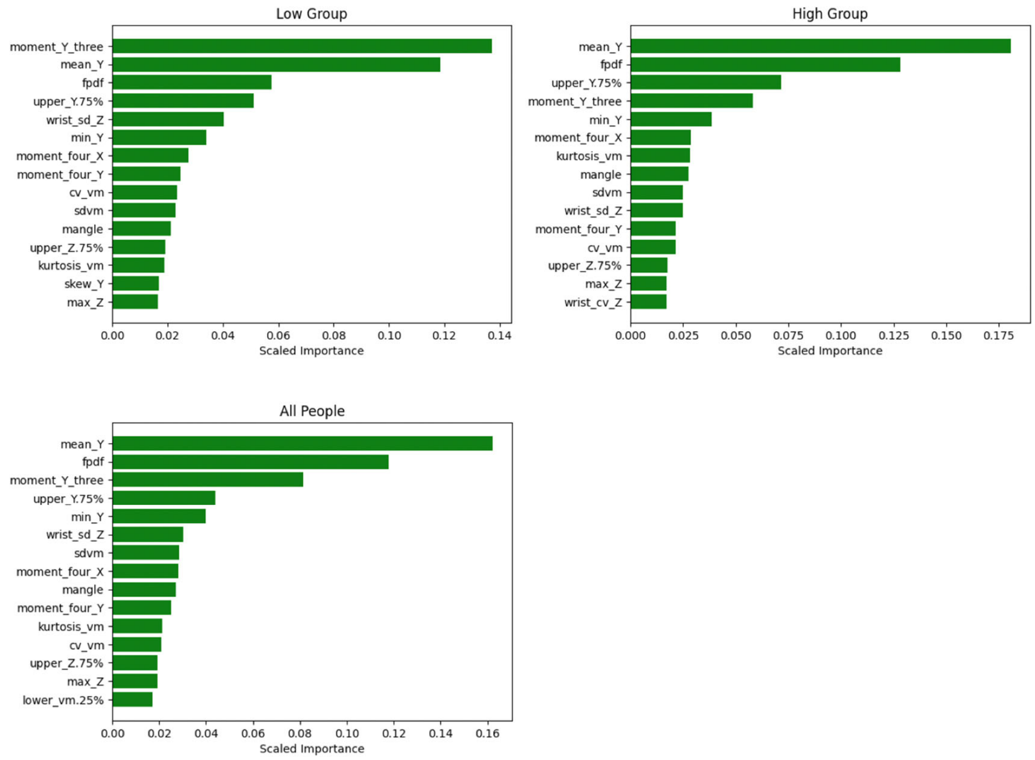

Figure S26. Feature importance for recognizing locomotion activities for different functioning groups.

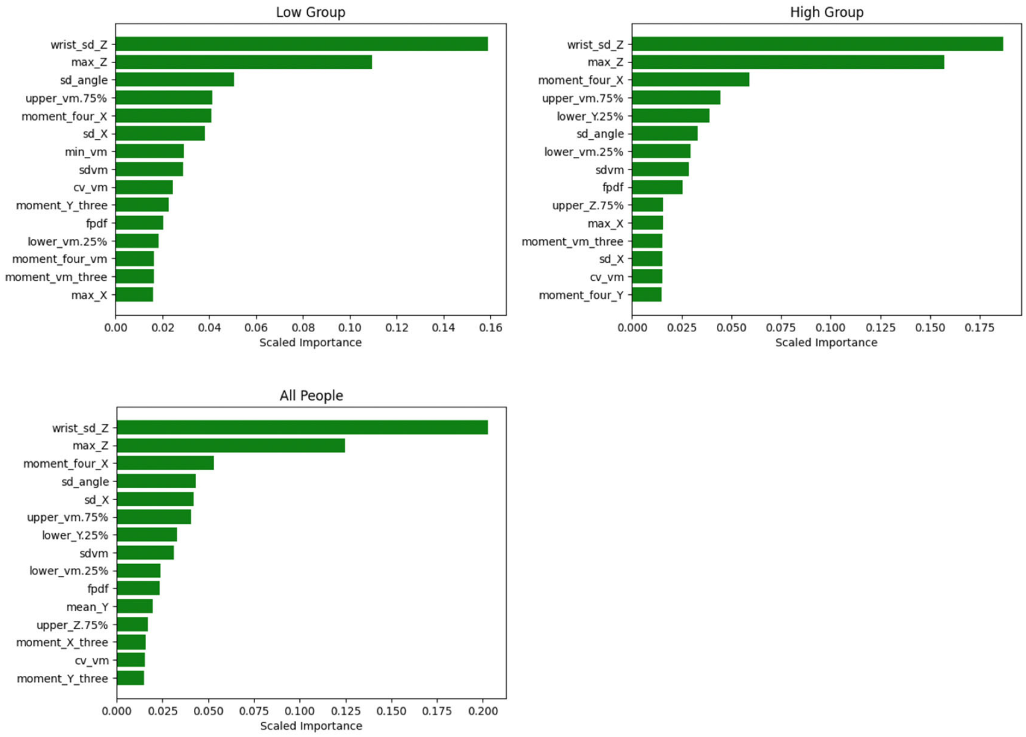

Figure S27. Feature importance for recognizing life-style activities for different functioning groups.

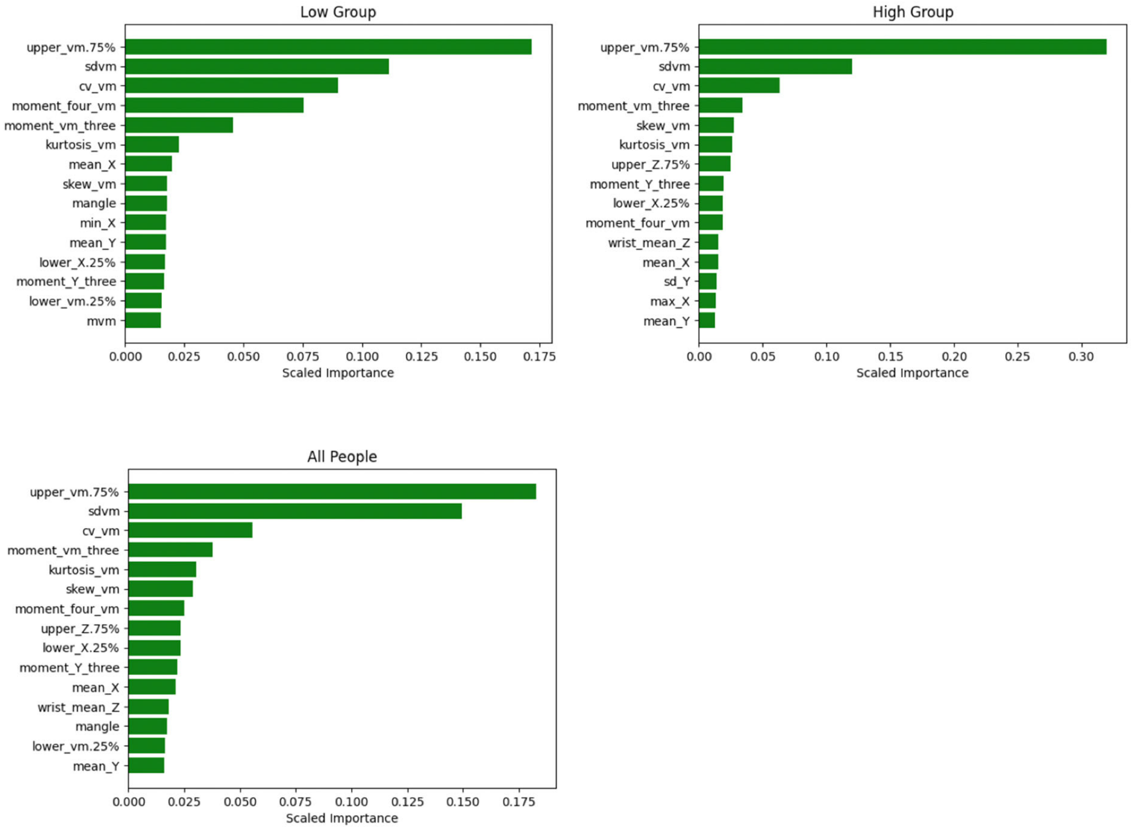

Figure S28. Feature importance for recognizing strength flexibility exercise activities for different functioning groups.

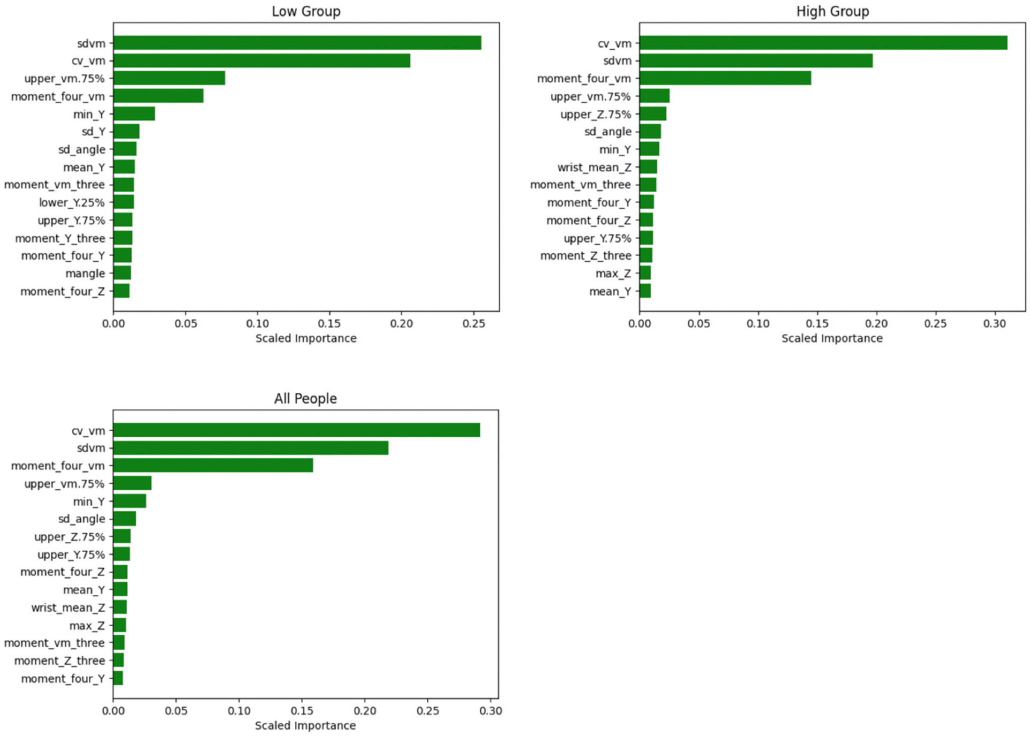

Figure S29. Feature importance for recognizing low intensity activities for different functioning groups.

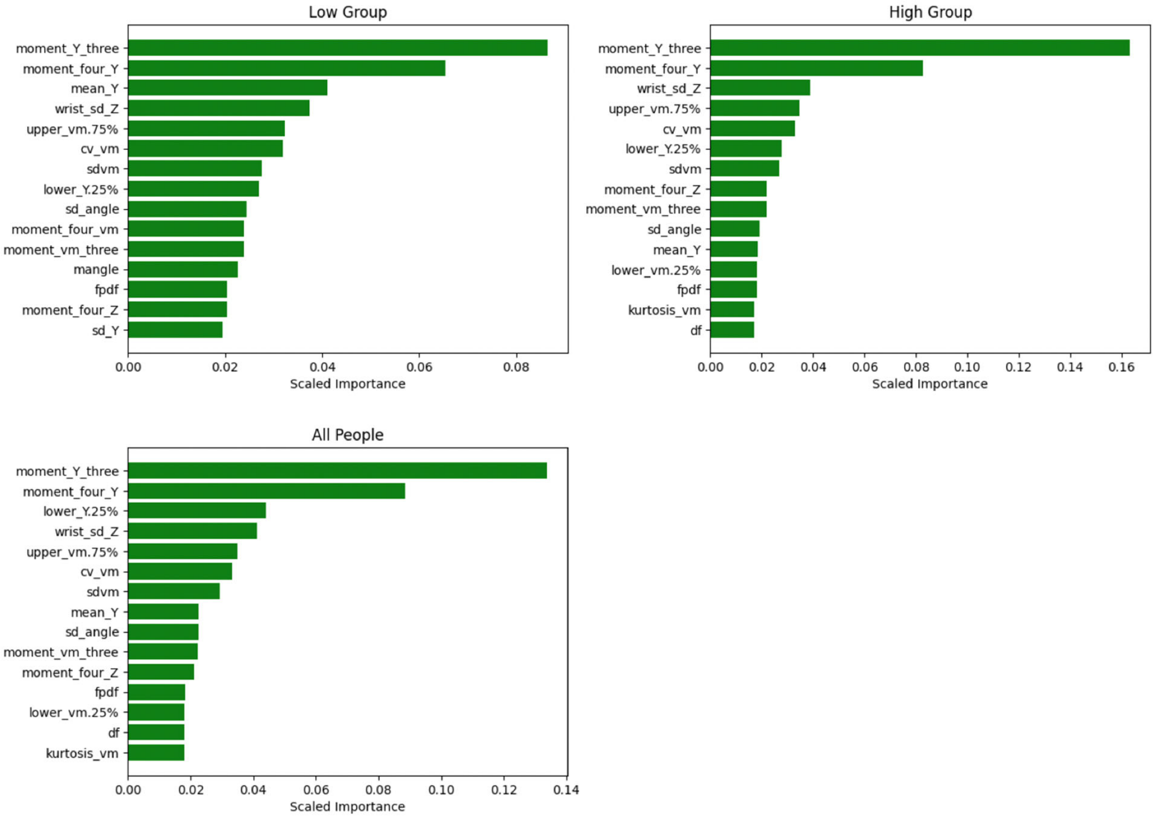

Figure S30. Feature importance for recognizing light intensity activities for different functioning groups.

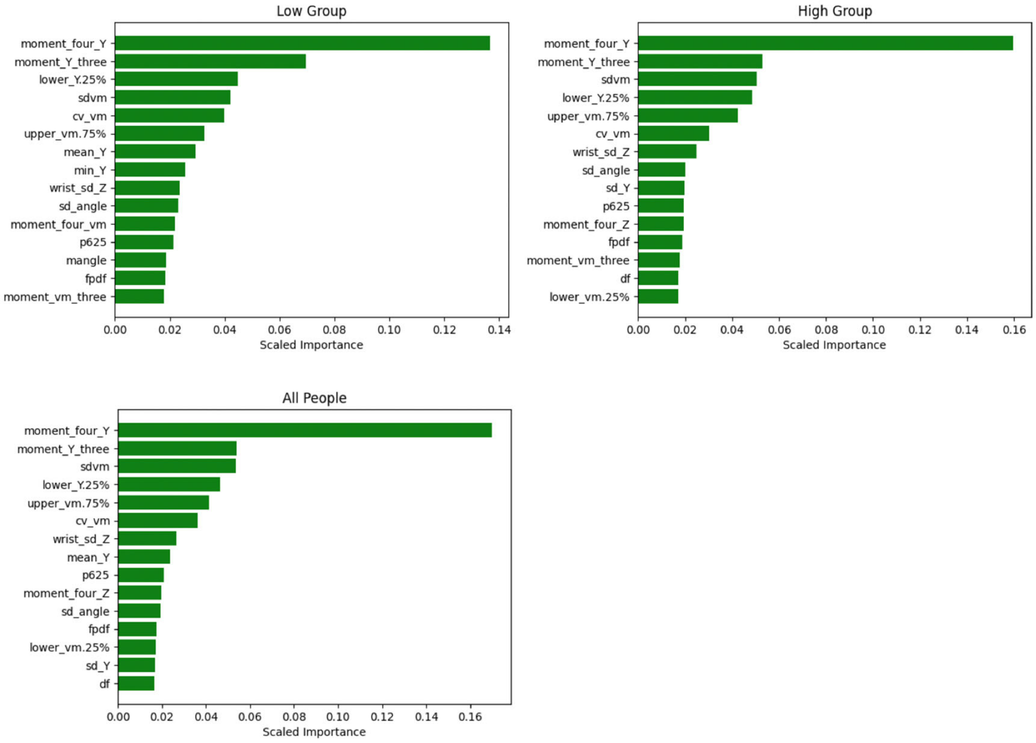

Figure S31. Feature importance for recognizing moderate intensity activities for different functioning groups.

Table S1. Comparison with relevant work in the literature. The listed studies collected from the wrist position for physical activity type, physical activity intensity and individual activity recognition. classification performance is accuracy unless otherwise stated. For the purpose of comparison, we calculated the average performance for the recognition task (i.e. physical activity type recognition). N is the number of participants; Num is number; ML is machine learning; RMSE is the root mean square error; RF is random forest; SVM is support vector machine; DT is decision tree and ANN is artificial neural network.

Reference	N	Age	Recognition Task	Num Activities	Num Categories	ML Method	Classification Performance	RMSE
Our work	247	72.4 ± 7.1	PA type recognition	33	4	XGBoost	92.4% ± 0.05	0.836 ± 0.059
Ellis et al. [49]	40	35.8 ± 12.1	PA type recognition	8	4 and 8	RF	87.5% and 80.2%	1.00
Chernbumroong et al. [2]	7	27-35	PA type recognition	5	5	DT and ANN	94.13%	NA
Staudenmayer et al. [32]	20	24.1 ± 4.5	PA type recognition	19	2 and 3	RF	96-99% and 76%	1.21
Davoudi et al. [14]	40	55.2 ± 17.8	PA type and intensity recognition	15	3	Multiple	87% (PA intensity), 100% (locomotion) and 98% (sedentary)	0.71
Mannini et al. [1]	33	18-75	PA type recognition	26	4	SVM	84.7%	NA
Weiss et al. [3]	17	NA	Individual activity recognition	18	18	Multiple	59.3-70.3%	NA

Studies that did not clarify the distribution of the age of participants and did not measure oxygen consumption were labeled as "not applicable" (NA).

The accuracy reported for the study of Weiss et al. was obtained from their impersonal models where the model was built using training data from 16 participants and was tested on the remaining one. An accuracy of 86.1 - 94.2% was obtained from their personal model where the model was built and tested on data from one participant at a time.
